# Spatiotemporal Control of the Formation of Luminescent Lanthanide Complexes in Liposome‐Based Nanoreactors

**DOI:** 10.1002/anie.202510471

**Published:** 2025-09-13

**Authors:** Aaron Torres‐Huerta, Miriam de J. Velásquez‐Hernández, Elena Tamarit‐Amoros, Marina Raschetti, Daniel Pinkas, Ondřej Jurček, Javier Pérez, Hennie Valkenier

**Affiliations:** ^1^ Engineering of Molecular NanoSystems (EMNS) Université libre de Bruxelles Avenue F. Roosevelt 50, CP165/64 Brussels B‐1050 Belgium; ^2^ Center for Membrane Separations Adsorption Catalysis, and Spectroscopy (cMACS) KU Leuven Leuven 3001 Belgium; ^3^ CNRS, Institut FEMTO‐ST Université Marie et Louis Pasteur Besançon F‐25000 France; ^4^ CEITEC – Central European Institute of Technology Masaryk University Kamenice 5 Brno CZ‐62500 Czechia; ^5^ Department of Natural Drugs Faculty of Pharmacy Masaryk University Palackého 1946/1 Brno CZ‐61200 Czechia; ^6^ Synchrotron SOLEIL Gif‐sur‐Yvette F‐91192 France

**Keywords:** Ion transport, Lanthanide complexes, Liposomes, Nanoreactors, Nanostructures

## Abstract

The controlled mass transfer across compartmentalised environments in synthetic nanoreactors is essential for enhancing precise spatiotemporal manipulation of chemical transformations within confined spaces. In this study, we present a strategy that integrates a synthetic anion transporter in liposome‐based nanoreactors, allowing for spatiotemporal control over the formation of metal–organic complexes in liposomes. This approach enables us to effectively modulate the assembly of luminescent lanthanide‐benzenedicarboxylate nanostructures. Fluorescence studies demonstrate that the reaction rate can be customised by varying the anion transporter concentration, which dictates the rate of entry of the carboxylate ligand. Changes in the morphology of the liposome nanoreactors due to the assisted transmembrane transport of benzenedicarboxylate were investigated using cryo‐TEM and time‐resolved SAXS measurements, which revealed a structural transformation of the lipid bilayer during the complex formation. Our findings provide a novel platform for exploring coordination chemistry in nanoscale confinements, opening avenues for the design of biohybrid materials.

## Graphical abstract

Synthetic ion transporters enable precise control over carboxylate transport across liposome membranes that are preloaded with Ln^3+^ ions. This process triggers the spatiotemporal formation of luminescent lanthanide complexes within liposome‐based nanoreactors. Cryo‐TEM and small‐angle X‐ray scattering reveal that the complexation of lanthanides induces a structural transformation of liposomes from unilamellar to bilamellar vesicles.

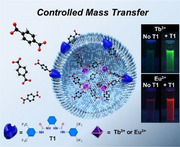

## Introduction

Biological cells are able to execute complex metabolic processes in a highly efficient and spatially controlled manner.^[^ ^1^, ^2^ ^]^ This capability arises from the compartmentalisation of chemical transformations within organelles.^[^ ^3^ ^]^ These compartments facilitate the formation of concentration gradients, enable the separation of chemical reactions and allow for the precise regulation of cascade catalytic transformations.^[^ ^4^ ^]^ Inspired by this natural compartmentalisation, significant efforts have been directed toward mimicking lipid membrane‐based compartmentalisation and processes related to the formation of these compartments in artificial systems,^[^ ^5^, ^6^, ^7^, ^8^, ^9^, ^10^ ^]^ as well as toward developing synthetic nanoreactors that mimic cellular architecture.^[^ ^11^ ^]^ These artificial nanoreactors are nanoscale compartments enclosed by boundary layers, where chemical reactions occur within confined volumes ranging from femtoliters to yoctoliters (10^−15^–10^−24^ L).^[^ ^12^ ^]^ By separating reaction spaces from the bulk environment, artificial nanoreactors can afford an enriched concentration of reactants, which enhances the probability of effective molecular collisions accelerating chemical reaction rates.^[^ ^12^, ^13^, ^14^ ^]^ Furthermore, artificial nanoreactors offer powerful synthetic platforms for templating nanoparticle formation as their confined volumes provide an isolated space for controlling particle nucleation and growth.^[^ ^12^, ^15^, ^16^ ^]^

To date, a wide variety of nano‐ and microscale structures have been utilised as artificial nanoreactors to emulate cellular compartmentalisation. These range from rigid inorganic matrices like porous silica^[^ ^17^ ^]^ to soft materials such as polymerosomes,^[^ ^18^ ^]^ micelles^[^ ^19^ ^]^ and liposomes.^[^ ^2^, ^19^ ^]^ Among these, liposomes stand out due to their structural and functional resemblance to natural bilayers.^[^ ^2^ ^]^ Liposomes are vesicular structures formed by the self‐assembly of amphiphilic phospholipids in aqueous solutions.^[^ ^20^ ^]^ These lipids feature hydrophilic polar heads that interact favourably with water, while the unfavourable interaction of the hydrophobic tails and water leads to a bilayer membrane formation, enclosing an aqueous core. The lipidic membrane allows for the passive diffusion of small molecules such as CO_2_, N_2_, O_2_ and ethanol, driven by concentration gradients.^[^ ^21^ ^]^ However, spontaneous diffusion alone is insufficient for the transmembrane transport of larger hydrophilic or charged molecules, substrates and cellular metabolites. For these species, internalisation across biological membranes can be achieved via transporter‐mediated processes.^[^ ^22^, ^23^ ^]^

Achieving controlled molecular transport in synthetic nanoreactors, a capability crucial for emulating biological systems, remains a significant challenge.^[^ ^12^ ^]^ Current artificial nanoreactors face severe mass transfer limitations due to the restricted permeability of their boundaries.^[^ ^14^ ^]^ These limitations hinder effective chemical communication between the compartmentalised nano environment and the external media, which is essential for regulating molecular enrichment, ion concentration gradients and spatiotemporal control over chemical transformations.^[^ ^12^, ^14^, ^24^ ^]^ These properties are highly sought after in applications like energy storage,^[^ ^20^ ^]^ catalysis,^[^ ^25^ ^]^ biomedical engineering^[^ ^26^ ^]^ and multicomponent nanoparticle synthesis.^[^ ^12^ ^]^ Therefore, developing nanoreactors with controlled and selective permeability is essential to advance precise spatiotemporal manipulation of chemical transformations within confined spaces.

To tackle this challenge and better emulate biological compartments, this study introduces the development of liposome‐based nanoreactors (LNRs) designed to facilitate the internalisation of charged organic molecules, assisted by synthetic anion transporters (i.e., anionophores) embedded in the lipid membrane.^[^ ^27^, ^28^ ^]^ Synthetic anion transporters so far have mainly been developed for fundamental studies or for biomedical applications.^[^ ^29^, ^30^, ^31^, ^32^, ^33^, ^34^ ^]^ Building upon these foundational contributions, our work leverages synthetic transmembrane transport of anions to expand our synthetic toolbox for preparing colloidal hybrid materials within compartmentalised nano‐environments.

**Figure 1:**
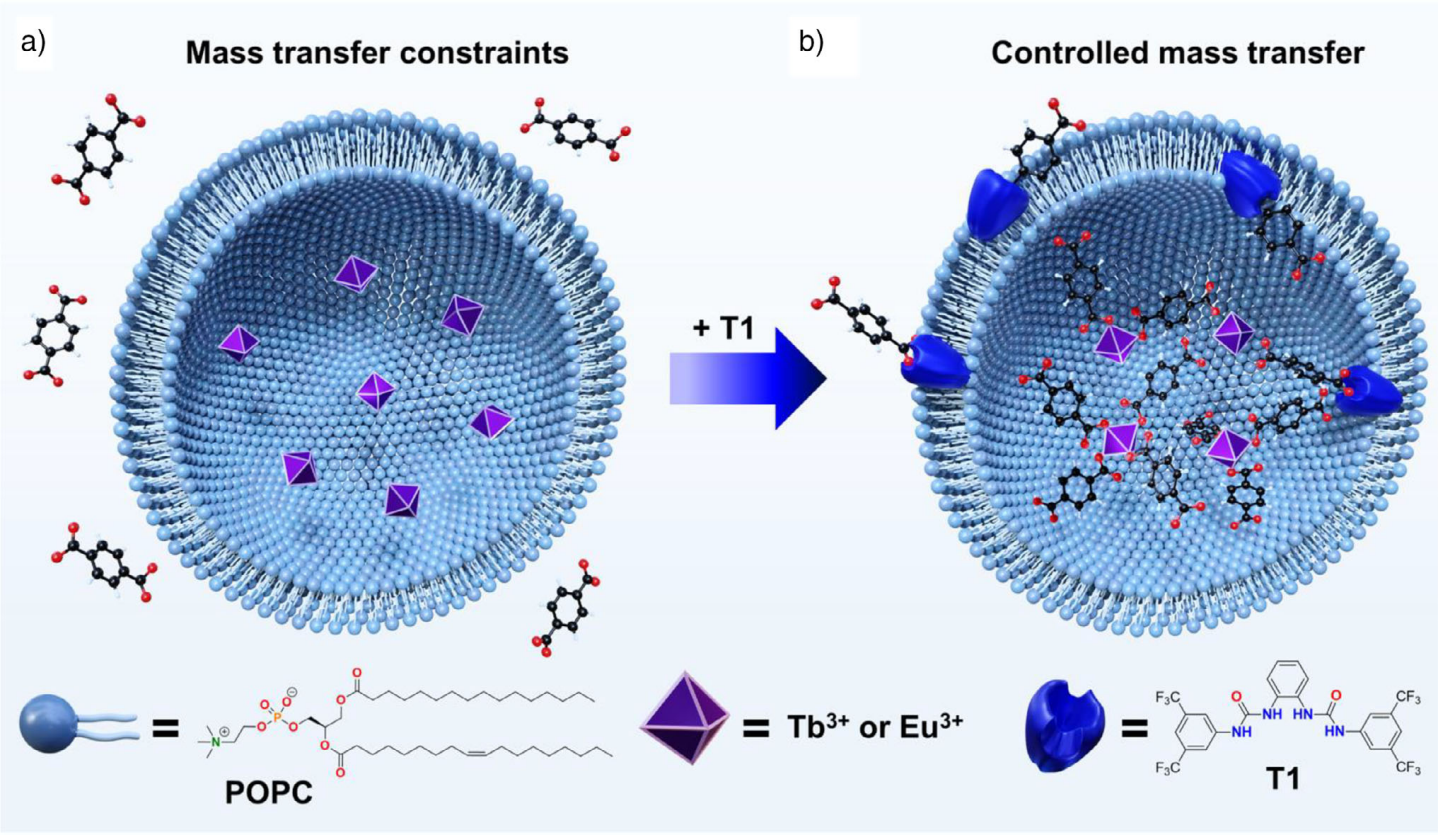
Schematic representation of liposome‐based nanoreactors. a) Artificial nanoreactor with mass transfer constraints imposed by the reactor boundaries. b) Artificial nanoreactor with anion transporter **T1** embedded in the lipidic bilayer to assist the transmembrane transport of benzene‐1,4‐dicarboxylate (BDC^2–^) to control the self‐assembly of metal‐organic complexes within confinement environments.

To evaluate the effectiveness of these nanoreactors in achieving efficient mass transfer, we used 200 nm liposomes preloaded with Ln^3+^ ions (Ln^3+^ = Tb^3+^, Eu^3+^), where the internalisation of benzene‐1,4‐dicarboxylate (BDC^2–^) is assisted by a bisurea‐based anion transporter **T1** (Figure 1). The suitability of this compound to assist the transmembrane transport of organic anions was previously reported.^[^ ^35^, ^36^, ^37^, ^38^ ^]^ However, there are no current reports where the assisted ion‐transport is exploited to control the spontaneous formation of coordination complexes within confinement environments. The successful transporter‐mediated internalisation of BDC^2–^ and its subsequent coordination with lanthanide (Ln^3+^) cations were monitored by emission spectroscopy as an increase in the emission intensity, indicating the formation of the LnBDC@LP colloidal system (Ln = Tb, Eu; LP = Liposome). The morphology changes of LNRs caused by the assisted BDC^2–^ transmembrane transport were investigated by cryo‐transmission electron microscopy (cryo‐TEM), whereas time‐resolved small‐angle X‐ray scattering (SAXS) was employed to monitor the lamellarity evolution of the lipid membrane during the BDC^2–^ transport.

## Results and Discussion

### Compartmentalisation of Ln^3+^ Cations Within Bioinspired Liposome‐Based Nanoreactors

Liposome‐based nanoreactors (LNRs) were prepared from the unsaturated lipid 1‐palmitoyl‐2‐oleoyl‐*sn*‐glycero‐3‐phosphocholine (POPC) mixed with cholesterol in a 7:3 molar ratio.^[^ ^39^ ^]^ The intravesicular cavity was loaded with LnCl_3_ by hydrating the lipid film with an aqueous solution of LnCl_3_ (7 mM), followed by freeze‐thaw cycles and extrusion using a polycarbonate membrane with a pore size of 200 nm to afford the desired LnCl_3_@LP vesicles (Figure 1a). Unencapsulated Ln^3+^ and Cl^–^ ions were removed by dialysis against a Na_2_SO_4_ solution (10.5 mM). The purified LnCl_3_@LP vesicles were suspended in Na_2_SO_4_ medium to achieve a final lipid concentration of 3 mM.

**Figure 2:**
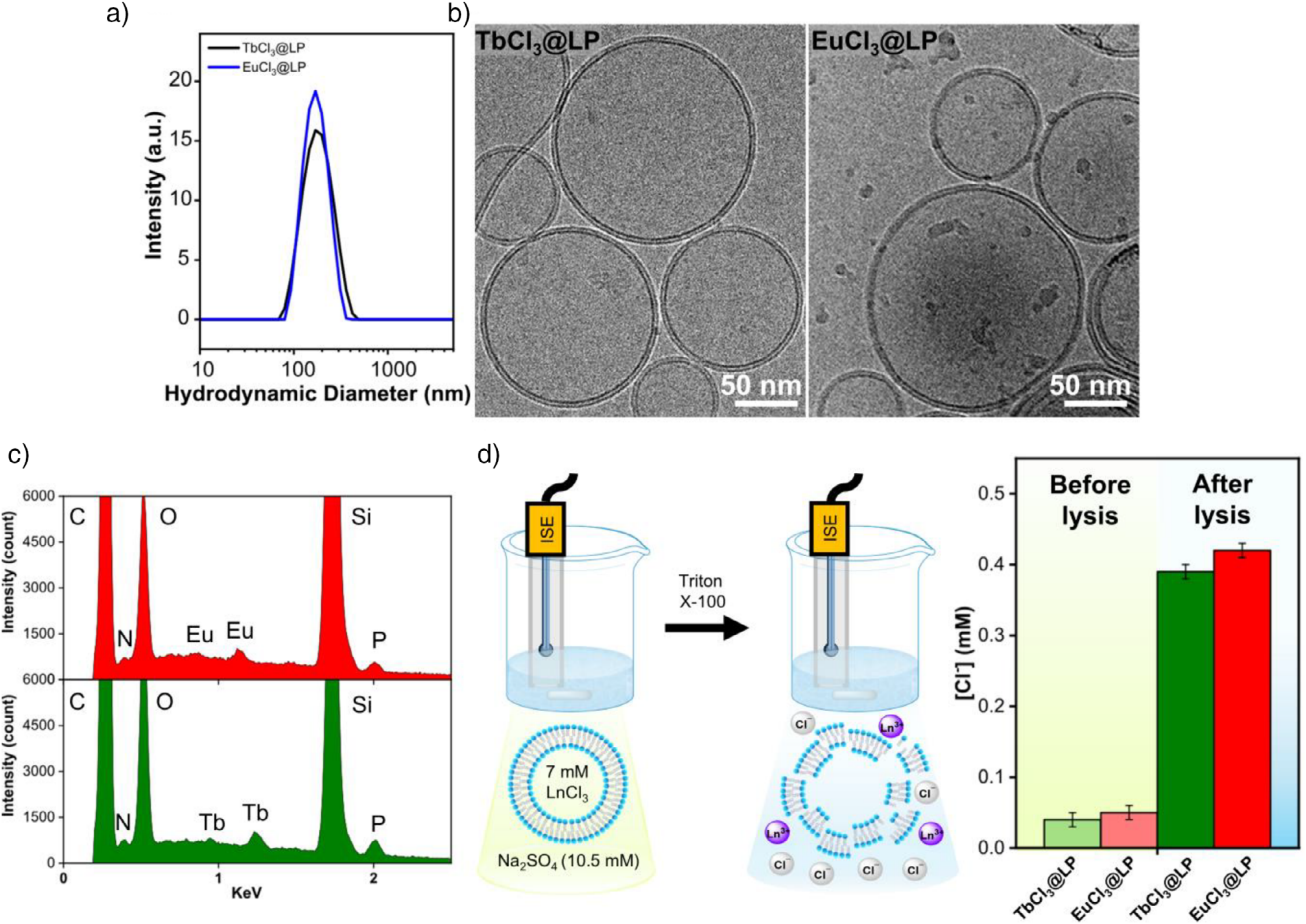
a) DLS and b) cryo‐TEM analysis for the TbCl_3_@LP and EuCl_3_@LP samples. c) EDX analysis confirms the co‐localisation of Tb^3+^ and Eu3^+^ in the corresponding LNRs. d) Schematic of Cl‐ISE assay to determine Cl^–^ concentration before and after lysing LnCl_3_@LP systems. Chloride concentrations obtained from 3 mL suspension of TbCl_3_@LP (green) and EuCl_3_@LP (red).

The resultant TbCl_3_@LP and EuCl_3_@LP suspensions exhibited high colloidal stability with a monodisperse size distribution as indicated in the dynamic light scattering (DLS) measurements, where the hydrodynamic diameter obtained for both samples was ca. 170 nm (Figure 2a). The formation of large unilamellar vesicles (LUVs) in the TbCl_3_@LP and EuCl_3_@LP suspensions was further confirmed by using cryo‐TEM analysis. The collected images revealed the formation of predominantly spherical unilamellar liposomes, identified by paired dark parallel lines, representing electron density variations within the bilayer membrane (Figure 2b). The average bilayer thickness measured for TbCl_3_@LP and EuCl_3_@LP samples was 5.3 ± 0.1 nm and 5.5 ± 0.1 nm, respectively.

The selective encapsulation of Tb^3+^ and Eu^3+^ within liposomes was initially assessed by using scanning electron microscopy coupled with energy‐dispersive X‐ray spectroscopy (SEM‐EDX, Figure S2). The elemental analysis collected from TbCl_3_@LP and EuCl_3_@LP samples, which were dried under vacuum, confirms the co‐localization of the corresponding lanthanide with the N and P associated with the POPC lipid (Figure 2c). To confirm the selective encapsulation of TbCl_3_ and EuCl_3_, as well as the effectiveness of dialysis in removing non‐encapsulated ions from the external environment (i.e., Tb^3+^, Eu^3+^ and Cl^–^), we used a chloride ion‐selective electrode (Cl‐ISE) to measure the Cl^–^ concentration in the surrounding environment of 3 mL suspension of each LnCl_3_@LP sample.^[^ ^36^ ^]^ The Cl‐ISE measurements revealed a residual chloride concentration of 0.04 ± 0.01 mM for the TbCl_3_@LP suspension and 0.05 ± 0.01 mM for EuCl_3_@LP analogue. Following a pulse of Triton X‐100 to induce lysis of the liposomes and release Ln^3+^ and Cl^–^ ions into the bulk solution, the chloride concentrations increased to 0.38 ± 0.01 mM and 0.42 ± 0.01 mM for TbCl_3_@LP and EuCl_3_@LP, respectively (Figures 2d and S1).

### Mass Transfer Control of BDC^2–^ to Yield Luminescent LnBDC@LP Nanosystems

To validate our hypothesis of using synthetic ion transporters to effectively regulate the mass transfer of molecular building blocks and achieve spatiotemporal control over the formation of metal–organic complexes inside liposomes, we developed an assay leveraging the luminescence properties of lanthanides when coordinated with carboxylate ligands. This assay monitors the controlled transport of BDC^2–^ (60 µL, 50 mM) into liposomes preloaded with Ln^3+^ by tracking the emission intensity of the Ln(BDC) complex. The ion‐transport test was conducted by adding transporter **T1** (3.3 µL of a 2.7 mM solution in methanol) to an aqueous LnCl_3_@LP suspension (3 mL), keeping a transporter‐to‐lipid ratio of 1:1000. The hydrophobic nature of **T1** promotes its integration into the lipidic membrane, initiating the dissipation of the BDC^2–^ gradient through a BDC^2–^/Cl^–^ ion exchange (Figure 1b).^[^ ^36^ ^]^ The urea moieties present in **T1** function as hydrogen bond donors, interacting with the negative charged oxygen atoms of the carboxylate group. The internalisation of BDC^2–^ is accompanied by a concomitant increase in emission, indicative of LnBDC@LP formation.

**Figure 3:**
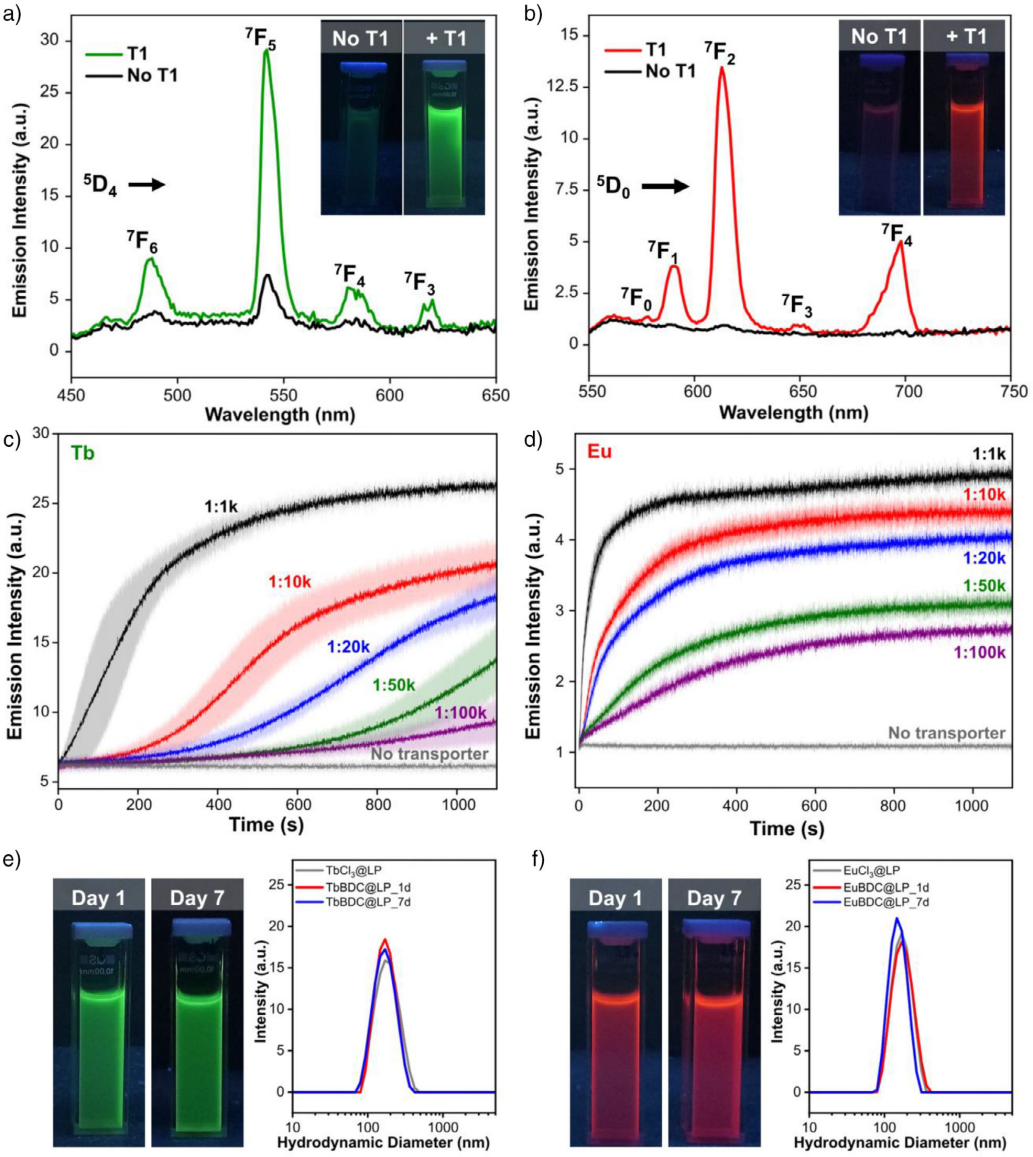
a) Comparative emission spectra for TbBDC@LP and the control sample (TbCl_3_@LP + BDC^2–^) when excited at *λ*_ex_ = 254 nm. Spectra were collected 1h after the addition of **T1** and BDC^2–^. Inset: photographs of colloidal liposome suspensions observed under a UV lamp. b) Emission spectra of EuBDC@LP alongside the control sample (EuCl_3_@LP + BDC^2–^) when excited at *λ*_ex_ = 312 nm. Spectra were collected 1h after the addition of **T1** and BDC^2–^. Inset: photographs of colloidal liposome suspensions observed under UV lamp. c) and d) The emission intensity of LnBDC@LP over time at different transporter‐to‐lipid ratios (Tb; *λ*_em_ = 542 nm, Eu; *λ*_em_ = 614 nm). e) and f) DLS analysis of LnBDC@LP samples collected 1 and 7 days after the addition of **T1** and BDC^2–^. Photographs of the colloidal liposome suspensions under UV lamp.

The emission spectra collected from the TbBDC@LP sample revealed the characteristics terbium transitions ^5^D_4_→^7^F_j_ (j = 6, 5, 4, 3) upon excitation at *λ*_ex_ = 254 nm, consistent with carboxylate coordination^[^ ^40^, ^41^ ^]^ (Figure S3). The strongest emission was observed at 542 nm, corresponding to the ^5^D_4_→⁷F_5_ transition (Figure 3a). Notably, the control sample TbCl_3_@LP in absence of **T1** exhibited significantly lower emission intensity upon BDC^2–^ addition. These results highlight the effectiveness of the liposomal membrane in isolating reactants and the critical role of **T1** to assist the BDC^2–^ internalisation. Additionally, control samples i) TbCl_3_@LP, ii) TbCl_3_@LP + **T1** and iii) NaCl@LP + **T1** + BDC^2–^ do not exhibit any emission, which confirms that the emission arises exclusively from the coordination of terbium with BDC^2–^ (Figure S4). The emission spectrum collected from EuBDC@LP exhibited the characteristic europium transitions ^5^D_0_→^7^F_j_ (j = 0–4) when exciting at *λ*_ex_ = 312 nm,^[^ ^41^ ^]^ with the strongest emission at 614 nm corresponding to the ^5^D_0_→^7^F_2_ transition (Figure 3b).

Following these results, we assessed the kinetics of BDC^2–^ transport and its subsequent coordination with Ln^3+^ ions by monitoring the emission over 20 min. Specifically, for TbBDC@LP samples, the kinetic assessment was performed by monitoring the intensity of the emission band at 542 nm (^5^D_4_→^7^F_5_). The dependence of the BDC^2–^ transport rate as a function of the transporter concentration was explored by adjusting the transporter‐to‐lipid ratio to 1:1000 (1:1 k), 1:10000 (1:10 k), 1:20000 (1:20 k), 1:50000 (1:50 k) and 1:100000 (1:100 k) (Figure 3c). The kinetic experiments revealed that at a transporter‐to‐lipid ratio of 1:1 k, the BDC^2–^ transport and subsequent coordination with the encapsulated Tb^3+^ ions reached a plateau in ca. 600 s, indicating the depletion of available Tb^3+^ ions for Tb(BDC) formation. In contrast, the experiments performed at lower transporter‐to‐lipid ratios (1:10 k, 1:20 k, 1:50 k and 1:100 k) exhibited slower rates of Ln(BDC) formation. These results confirm that the spatiotemporal control over the Ln(BDC) formation can be finely tuned by varying the transporter concentration. Notably, in the absence of **T1**, the emission intensity of TbCl_3_@LP in the presence of BDC^2–^ remained unchanged throughout the monitoring period. This observation confirms the absence of Tb^3+^ leakage or spontaneous diffusion of BDC^2–^ across the membranes (Figures 3a and S5).

A similar trend was observed for the EuBDC@LP samples, where a decrease of the transporter:lipid ratios resulted in slower BDC^2–^ transport rates (Figure 3d). However, for EuCl_3_@LP samples, a faster increase in emission intensity was observed compared to their TbCl_3_@LP analogues, under identical transporter concentrations. These results suggest that the coordination reaction between BDC^2–^ to Eu^3+^ proceeds faster than for Tb^3+^. This finding highlights the unique opportunities of our approach to evaluate the chemical reactivity of lanthanides in solution.

The DLS measurements collected from the resultant TbBDC@LP and EuBDC@LP suspensions indicate a monodisperse size distribution comparable to their precursors TbCl_3_@LP and EuCl_3_@LP. Interestingly, the products exhibited exceptional colloidal stability for at least seven days at room temperature (Figure 3e,f). These results underscore the protective role of liposomes in preventing aggregation of the colloidal systems. Furthermore, emission spectra collected from TbBDC@LP and EuBDC@LP after 1, 3 and 7 days did not show significant changes in the emission intensities, suggesting that the Ln(BDC) complexes remain chemically stable in aqueous media inside liposomes (Figures S6 and S14).

### Lamellarity Evolution of the Liposome‐Based Nanoreactors

**Figure 4:**
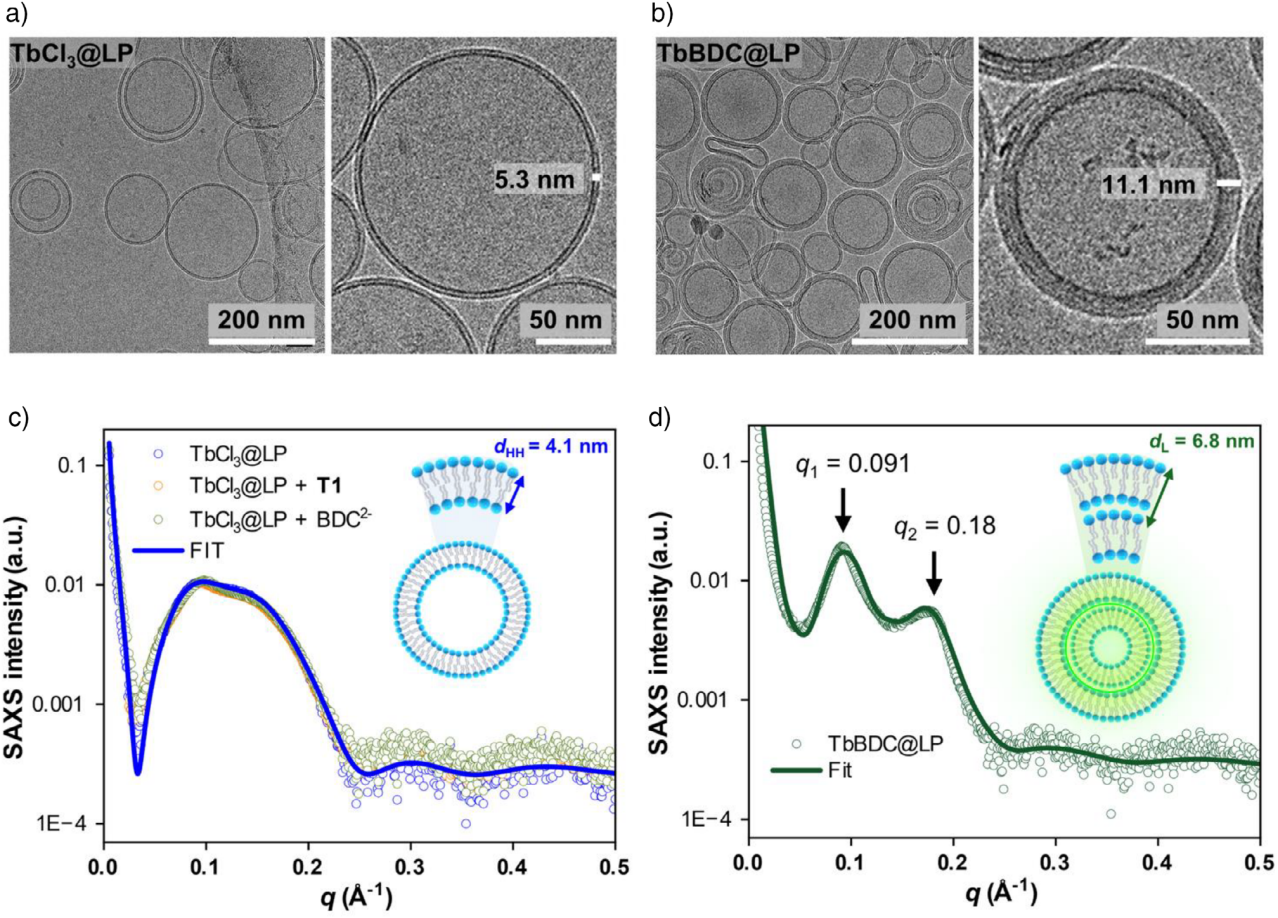
a) and b) Cryo‐TEM images of TbCl_3_@LP and TbBDC@LP. c) SAXS profiles of control samples: TbCl_3_@LP (blue; fitted), TbCl_3_@LP + **T1** (orange) and TbCl_3_@LP + BDC^2–^ (green). d) SAXS profile of TbBDC@LP.

Cryo‐TEM imaging and SAXS analysis were used to investigate the morphology of the TbBDC@LP samples. Cryo‐TEM images were collected for TbCl_3_@LP (control) and TbBDC@LP samples obtained by varying the transporter‐to‐lipid ratio (i.e., 1:10 k, 1:20 k, 1:50 k and 1:100 k) followed by the addition of BDC^2–^ pulse (1 mM). Conversely, to the unilamellar structures observed in the control sample TbCl_3_@LP (Figure 4a), the images collected from TbBDC@LP revealed the formation of bilamellar structures with a uniform thickness of 11.1 ± 0.1 nm (Figure 4b). Interestingly, such bilamellar structure consistently appeared in all TbBDC@LP samples independent of the transporter‐to‐lipid ratios tested (Figure S17–S21). This observation suggests that the final bilamellar arrangement is not affected by the BDC^2–^ transport rate. Additionally, the size distributions obtained from TbCl_3_@LP and TbBDC@LP samples revealed a decrease of the average diameter of the liposomes from 124 ± 43 to 105 ± 28 nm (Figure S16 and S17). Furthermore, the control sample NaCl@LP + **T1** + BDC^2–^ maintained a unilamellar morphology (Figure S22), confirming that the formation of bilamellar structures is a direct consequence of the Tb(BDC) complex formation inside the liposomes.

To get more insight into the multilamellar vesicle formation in the bulk TbBDC@LP sample, we conducted SAXS measurements using synchrotron radiation.^[^ ^42^ ^]^ To optimise the scattering intensity the lipid concentration was increased to 6.4 mM, while **T1** and BDC^2–^ concentrations were adjusted to match the molar ratios used in previous experiments. For each measurement, 7 mL of the liposome suspension was recirculated through a 1.5 mm thick quartz capillary at room temperature. The SAXS profiles were analysed using a model combining a Gaussian bilayer form factor and a modified Caillé structure factor,^[^ ^43^, ^44^ ^]^ which includes variables to quantify lamellarity, such as the number of layers (N). Details of the model and fitting procedure are provided in Section 8 of the Supplementary Information.

The experimental SAXS profile and fitting of *I*(*q*) versus *q* for the TbCl_3_@LP sample revealed that in the *q* region of the form factor associated with the bilayer structure (0.03 < *q* < 0.25), the scattering patterns exhibited a characteristic single bump, indicating the presence of predominantly unilamellar liposomes (Figure 4c).^[^ ^45^ ^]^ The best model description of the data was accomplished with lamellarity *N* = 1.1 and an estimated headgroup‐to‐headgroup thickness (*d*_HH_) of 4.1 nm (Figure S26, Table S1), which is consistent with prior reports for POPC:cholesterol liposomes.^[^ ^44^ ^]^ The SAXS profiles for TbCl_3_@LP samples revealed no significant structural modifications upon the individual addition of either **T1** or BDC^2–^. These findings confirm that neither **T1** nor BDC^2–^ alone drives significant structural changes in the lipid membrane. Additionally, these results indicate that the synthetic anion transporter **T1** does not compromise the integrity of the phospholipid bilayer, supporting the feasibility of carrier‐mediated transport to control the mass transfer of BDC^2–^ into the liposomes.

Contrary to TbCl_3_@LP, the scattering profile obtained from TbBDC@LP displayed two broad peaks centred at *q*_1_ = 0.091 and *q*_2_ = 0.180 Å^−1^, corresponding to the first and second order of Bragg reflections from a repeating layered structure (Figure 4d).^[^ ^45^ ^]^ This SAXS profile indicates the formation of multilamellar vesicles (MLVs) with a layer spacing (*d_L_*) of 6.8 nm. The Caillé structure factor was used to extract the number of repeating multilayers, where the obtained value was *N* = 2.3. Despite the lamellarity increase, the *d*_HH_ distance remained relatively consistent with a value of 4.05 nm (Table S1).

**Figure 5:**
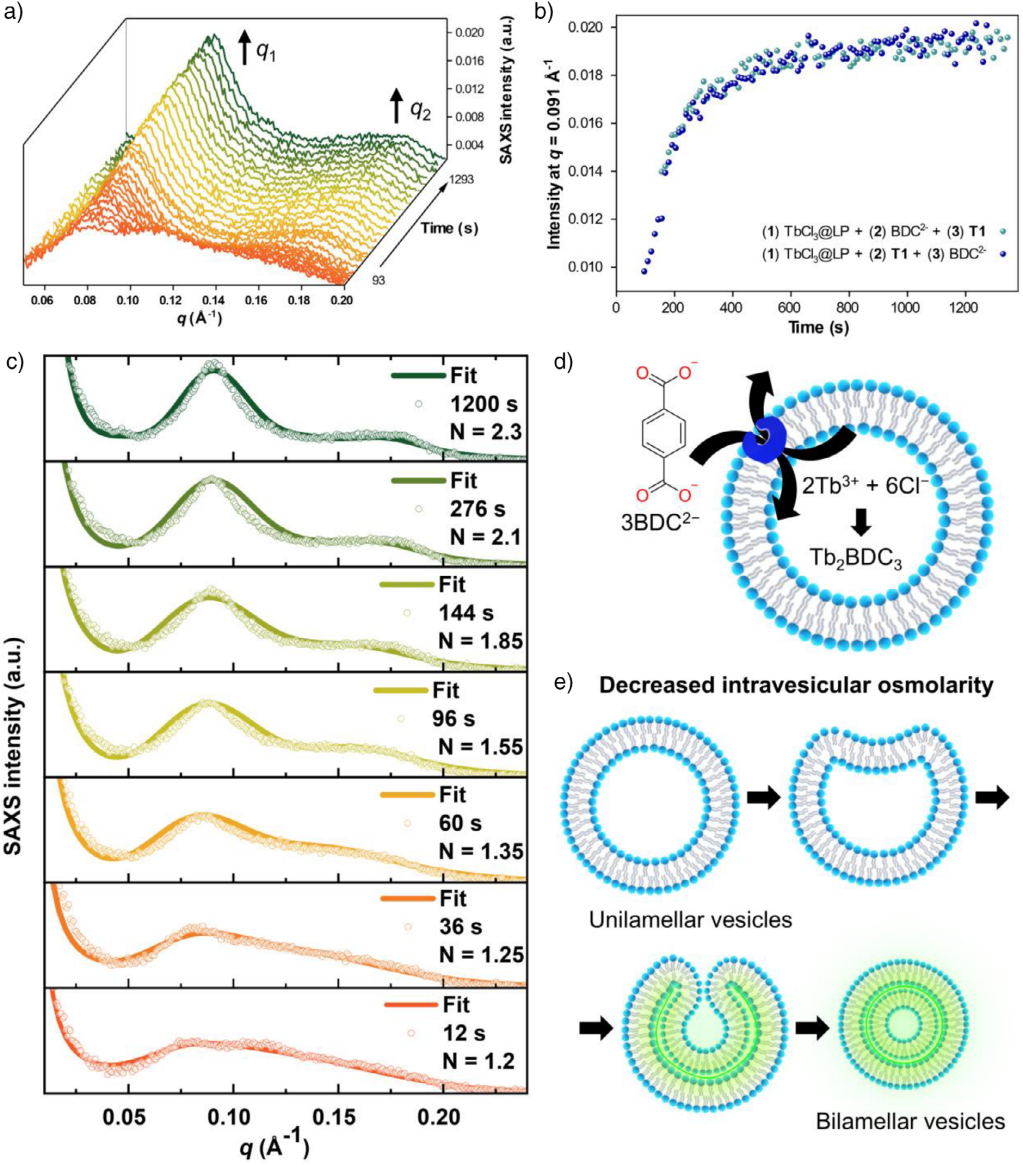
a) SAXS profile tracking the formation of TbBDC@LP over 20 min. Selected frames are shown for clarity, while full‐frame acquisitions are provided in Figures S28–S31. b) Evolution of *q*_1_ intensity over 20 min during the formation of TbBDC@LP. c) Fitting of selected SAXS frames illustrating the transition from unilamellar to bilamellar vesicles during TbBDC@LP formation. d) and e) Schematic representation of bilamellar vesicle formation induced by osmolarity reduction upon the anti‐port BDC^2–^ transport.

To assess the transformation from unilamellar to bilamellar vesicles upon BDC^2–^ transport, we performed time‐resolved SAXS measurements. The structural evolution of TbBDC@LP was analysed using a transporter‐to‐lipid ratio of 1:10 k. This approach leveraged the high brilliance of synchrotron X‐ray sources to monitor dynamic structural changes, within the time frame used in the emission experiments, collecting SAXS frames every 10 s for 20 min. The first frame, acquired at 93 s after the BDC^2–^ pulse, already displayed two broad, low‐intensity peaks at *q*_1_ = 0.091 Å^−1^ and *q*_2_ = 0.180 Å^−1^, indicative of bilamellar vesicle formation. The intensity of these peaks increased over time, reaching a plateau within 600 s (Figure 5a). Notably, the time frame in which the most significant morphological changes occurred closely aligns with the variations in emission intensity observed at the same **T1** concentration (1:10 k, Figure 3c). This correlation strongly suggests that the formation of the bilamellar structure is directly linked to the formation of the Tb(BDC) complex. Interestingly, the rate of *q*_1_ intensity evolution was unaffected by the sequence of component addition–whether **T1** was added first to TbCl_3_@LP suspension followed by BDC^2–^, or vice versa (Figure 5b). However, a decrease in **T1** concentration resulted in a slower *q*_1_ intensity evolution (Figure S32). Fitting selected SAXS profiles using the Gaussian bilayer form factor and Caillé structure factor,^[^ ^43^, ^44^ ^]^ indicated a progressive increase in lamellarity, from *N* = 1.2 to a maximum of *N* = 2.3 (Figure 5c). This evolution was accompanied by a decrease in layer spacing (*d*_L_) from 7.5 to 6.8 nm, while the headgroup‐to‐headgroup distance (*d*_HH_) remained constant within the range of 3.9–4.1 nm (Table S1).

The unilamellar‐to‐bilamellar transition observed in both cryo‐TEM and SAXS analyses may be attributed to osmotic pressure variations between the bulk solution and the internal liposomal cavity during TbBDC@LP formation.^[^ ^46^, ^47^ ^]^ This osmotic shrinkage is likely induced by two key processes: i) the exchange of one BDC^2–^ anions for two Cl^–^ ions via an antiport mechanism to avoid the buildup of an electrostatic potential^[^ ^36^ ^]^ and ii) the coordination of the BDC^2–^ with Tb^3+^ cations inside the liposome (Figure 5d). The combination of these processes induces osmotic stress, driving the inward folding of the lipid bilayer and the subsequent formation of bilamellar vesicles (Figure 5e). This hypothesis is consistent by the decrease in vesicle radius observed in the cryo‐TEM images for TbBDC@LP with respect to TbCl_3_@LP (see Supplementary Information, section 7). Interestingly, these changes were not reflected in DLS measurements as this technique provides hydrodynamic diameters rather than the actual vesicle dimensions observed in cryo‐TEM images.

Although bilamellar vesicle formation induced by the hypertonic osmotic shock in saline solutions has been previously reported,^[^ ^48^ ^]^ this study presents the first example of multilamellar vesicle formation triggered by the assembly of a metal–organic complex within liposomes and resulting in bilamellar vesicles with a homogeneous membrane thickness. We note that his process can take place in liposomes of different lipid compositions (see Figures S11 and S12 for experiments using POPC and DOPC, respectively) and that similar bilamellar vesicles were obtained when using succinate instead of BDC^2–^ (Figure S24). The controlled formation of artificial bilamellar vesicles provides a platform to mimic processes like endocytosis, as well as biological double‐membrane structures, such as those found in organelles and Gram‐negative bacteria. Accordingly, this system offers a suitable model for studying structural protection, selective permeability and antibiotic resistance mechanisms associated with these biological membranes.

**Figure 6:**
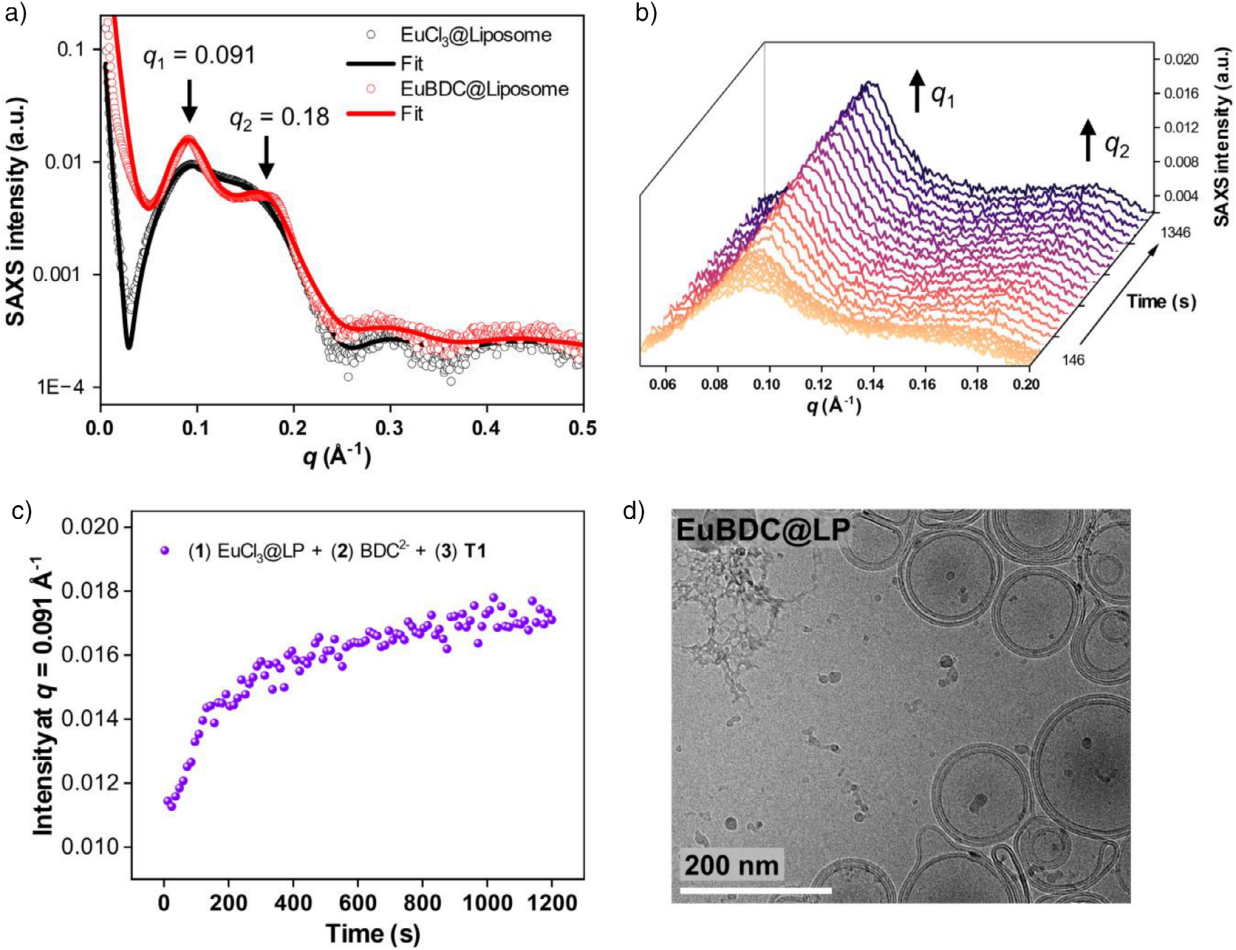
a) SAXS profiles and fitting of liposomes: EuCl_3_@LP (black) and EuBDC@LP (red). b) Time‐resolved SAXS measurements tracking the formation of EuBDC@LP over 20 min. Selected frames are shown. Full‐frame acquisitions are presented in Figure S33. c) Evolution of *q_1_* intensity over 20 min during the formation of EuBDC@LP. d) Cryo‐TEM images of EuBDC@LP.

SAXS measurements were also conducted on EuBDC@LP and EuCl_3_@LP samples under the same experimental conditions used for their terbium‐based counterparts. The SAXS profile of EuCl_3_@LP indicated the formation of predominantly unilamellar liposomes (*N* = 1.13). In contrast, the SAXS pattern for the final EuBDC@LP product displayed distinct peaks characteristic of multilamellar vesicles at *q*_1_ = 0.091 Å^−1^ and *q*_2_ = 0.180 Å^−1^ (*N* = 2.2) (Figure 6a). Although this pattern was similar to that observed in TbBDC@LP, the *q*_1_ and *q*_2_ peaks in the EuBDC@LP sample were slightly broader. Additionally, time‐resolved SAXS analysis of EuBDC@LP showed a rapid increase in the intensity of *q*_1_ immediately after the BDC^2–^ addition (Figure 6b). However, the intensity changes of *I*(*q*_1_) and *I*(*q*_2_) for the EuBDC@LP sample were less pronounced than that those observed for TbBDC@LP (Figure 6c). This could be attributed to the formation of less ordered lamellar structures, indicating less regular lamellar spacing in the EuBDC@LP sample. The presence of these low‐ordered lamellar structures was further confirmed by cryo‐TEM imaging (Figures 6d and S23).

## Conclusion

This study demonstrates that integrating synthetic transporters in the membranes of liposome‐based nanoreactors provides spatiotemporal control over the assembly of metal–organic complexes within compartmentalised nano‐environments. Our approach is underpinned by the supramolecular interactions between the transporter and the selected organic building block, facilitating its selective uptake and controlled internalisation within the nanoreactors. This transport mechanism establishes a dynamic exchange between the confined nano‐environment and the external medium while maintaining the permeability and protective function of the lipidic boundaries, as confirmed by SAXS and cryo‐TEM analyses.

For the first time, this strategy has been implemented to synthesise LnBDC@LP luminescent nanostructures, which exhibit, in contrast to LnBDC MOFs, long‐term colloidal and chemical stability. Emission studies reveal that the rate of Ln(BDC) formation can be tuned by varying the transporter‐to‐lipid ratio, while time‐resolved SAXS measurements indicate a structural transition of the lipid bilayer from unilamellar to bilamellar vesicles upon the Ln(BDC) formation. These results underscore the importance of thoroughly characterising nanoreactors to assess potential interactions between the confined products and the surrounding boundaries, particularly in systems based on soft materials. Moreover, our findings open exciting opportunities for investigating the early stages of coordination reactions and understanding how fundamental principles—such as nucleation, reactivity and size‐ or shape‐dependent properties—change in confined nano‐environments.

We anticipate that our approach can be extended to the synthesis of a wide range of hybrid nanomaterials, including multivariate complexes, where the stoichiometric composition of metal ions within liposomes can be precisely customised. Furthermore, we envision that LnBDC@LP nanosystems will serve as a promising platform for developing biomimetic materials for bioimaging and sensing applications, leveraging their luminescent properties and exceptional colloidal stability.

This work expands the synthetic toolbox for preparing colloidal supramolecular complexes in aqueous media by enabling the formation of highly reproducible nanoparticles with tight size control. The lipid membrane not only prevents particle agglomeration and inhibits chemical etching but also offers a versatile scaffold for tuning nanoparticle composition. Moreover, the straightforward functionalisation of the lipidic bilayer allows for customisation of membrane properties, opening avenues for targeted applications in catalysis, drug delivery and adaptive materials.

## Supporting Information

Experimental procedures, nanosystems characterization and data analysis. The authors have cited additional references within the Supporting Information.^[^ ^49^ ^]^

## Conflict of Interests

The authors declare no conflict of interest.

## Supporting information



Supporting Information

## Data Availability

The data that support the findings of this study are available in the Supporting Information of this article.
