# Supplementary material for: Spatiotemporal Control of the Formation of Luminescent Lanthanide Complexes in Liposome‐Based Nanoreactors
Source: Angew Chem Int Ed Engl. 2025 Sep 13;64(45):e202510471. doi: 10.1002/anie.202510471 (PMC12582006; doi:10.1002/anie.202510471)
Supplement: Supplementary file 1 — Supporting Information [file ANIE-64-e202510471-s001.pdf]

Contents

1. General experimental information 1
2. Chloride content inside liposomes 4
3. SEM-EDX analysis 5
4. Fluorescence studies 5
5. FT-IR spectroscopy 13
6. Cryo-TEM analysis 14
7. Estimation of the size of bilamellar vesicles 18
8. Synchrotron small-angle X-ray scattering (SAXS) measurements 20

1. General experimental information

The reagents and solvents used in this study were obtained from Sigma Aldrich and used without further purification unless otherwise specified. 1-palmitoyl-2-oleoyl-sn-glycero-3-phosphocholine (POPC) and cholesterol were purchased from Sigma Aldrich and Acros, respectively. Chloroform was deacidified before preparing lipid solutions by passing it through a column containing basic alumina. POPC solutions were stored at -20 °C, while cholesterol solutions were freshly prepared. All aqueous solutions were prepared using deionized water passed through a Millipore filtration system.
Dynamic Light Scattering (DLS) measurements were performed on a Malvern Zetasizer Ultra at 25 °C using disposable cuvettes. A standard refractive index of 1.45 for liposome solutions was applied to determine the average size and size distribution.

1.1 $LnCl_3$@LP preparation

Liposomes were prepared from a lipid mixture of 1-palmitoyl-2-oleoyl-*sn*-glycero-3-phosphocholine (POPC) and cholesterol at a 7:3 molar ratio in deacidified chloroform. The amounts of the lipids were calculated to achieve a final concentration of 3 mM (POPC + cholesterol). Chloroform was evaporated under a gentle stream of nitrogen, and the resulting lipid film was dried under vacuum overnight to ensure complete removal of the solvent.
The dried lipid film was rehydrated with 500 µL of 7 mM $LnCl_3$ (Ln = Tb, Eu) in Milli-Q water. The rehydrated mixture was sonicated for 1 minute and then stirred for 1 hour at room temperature. To convert the heterogeneous multilamellar vesicles into predominantly unilamellar vesicles, the suspension underwent 10 freeze-thaw cycles and was extruded 29 times through polycarbonate membranes with a pore size of 200 nm at room temperature.
The excess $LnCl_3$ outside the liposomes was removed by dialysis using Biotech CE Tubing dialysis membranes (MWCO 20 kDa) against an external solution of 10.5 mM Na_2SO_4. The final $LnCl_3$@LP solution was subsequently diluted with the external solution to achieve a lipid concentration of 3 mM, assuming there was no lipid loss during the preparation and purification processes.

1.2 Chloride content inside liposomes

The chloride content outside the liposomes was measured using a Fisherbrand Accument AB250 chloride ion-selective electrode, calibrated prior to each experiment. For this assay, 3

mL of the corresponding $LnCl_3$@LP solution was transferred to a 25 mL beaker with moderate stirring, and the electrode was immersed in the solution. Once the readings stabilized, they were recorded as the external chloride concentration $[Cl^-]_{ext}$. To determine the chloride content inside the liposomes, 100 μL of Triton X-100 (5% w/w in water) was added to lyse the liposomes, and the resulting measurements was recorded as the total chloride concentration $[Cl^-]_{global}$. Additionally, we evaluated the behaviour of BDC^{2-} without the assistance of **T1** using the Cl-ISE assay over 20 minutes. Negligible chloride efflux was observed following the addition of the BDC^{2-} pulse (Figure S1b and S1c), confirming that the liposomes remained stable. This indicated that BDC^{2-} does not disrupt the lipid membrane to cause chloride leakage and that the BDC^{2-}/chloride exchange relies on the presence of **T1** to proceed effectively.

The electrode was calibrated against $NaNO_3$ (0.5 M) solutions containing sodium chloride at concentrations of 1 ppm, 10 ppm, 100 ppm, and 1000 ppm.

1.3 SEM-EDX analysis

Samples were analysed using an APREO S Thermo Fisher SEM equipped with a set of Trinity detectors along an A-tube biased at 8 kV. The T1 detector captured the intense signal of high-energy backscattered electrons, while the T2 detector focused on secondary electrons that did not have sufficient energy to reach the T1 detector. The T3 detector captured secondary electrons with even lower energy (<2 eV).

For sample preparation, a small drop of the liposomal solution was deposited onto a silicon chip and dried under vacuum. The dried samples were carefully rinsed with deionised water to remove excess Na_2SO_4 and residual reactants external to the liposomes. After rinsing, the samples were dried again under vacuum. Subsequently, the silicon chip with the sample was coated with a thin carbon layer (<5 nm) to ensure electrical conductivity for SEM imaging. EDX measurements were performed on at least three different liposomes to ensure representativity.

1.4 Emission spectroscopy

Emission measurements were conducted using a Fluoromax-4 spectrometer with a quartz cuvette containing a magnetic stir bar. The cuvette holder's temperature was regulated at 25 °C using a water bath, and the temperature of the sample compartment was stabilised at 25 °C for 3 minutes before each experiment. For terbium-based samples, emission spectra were recorded from 450 nm to 650 nm using an excitation wavelength of 254 nm with a 450 nm long pass filter. For europium-based samples, spectra were collected from 550 nm to 750 using an excitation wavelength of 312 nm with a 550 nm long-pass filter.

To 3 mL of a $TbCl_3$@LP solution, **T1** dissolved in methanol (3.3 μL) was added at the specified transporter-to-lipid ratio. One minute after the start of the emission measurement, 60 μL of a 50 mM BDC^{2-} solution was added, resulting in a final BDC^{2-} concentration of 1 mM. The emission intensities at 542 nm and 614 nm were monitored over 20 minutes for terbium- and europium-based samples, respectively.

To assess long-term stability, the cuvettes were stored at room temperature for 7 days to monitor any changes in emission intensity. Photographs of the cuvettes were taken using a Samsung A4 smartphone under illumination from a handheld UV lamp (254 nm) for both TbBDC@LP and EuBDC@LP samples. All experiments were conducted in triplicate, and the emission data were averaged.

1.5 Cryo-TEM analysis

For the acquisition of cryo-TEM images, six fresh batches of TbBDC@LP were prepared, using **T1** in a transporter:lipid ratio of 1:1k, 1:10k, 1:20k, 1:50k, and 1:100k. 4 hours after the preparation of the TbBDC@LP samples, 4 µL of each sample was placed onto a freshly plasma-cleaned TEM grids (Quantifoil, Cu, 300 mesh, R2/1) and vitrified into liquid ethane using ThermoScientific Vitrobot Mark IV (4 °C, 100% rel. humidity, 30s waiting time, 6s blotting time). The grids were subsequently mounted into the Autogrid cartridges and loaded to Talos Arctica (ThermoScientific) transmission electron microscope for imaging. The microscope was operated at 200 kV. Images were collected on Ametek K2 or Falcon 4i direct electron detection camera at the 92000x nominal magnification with the underfocus in the range 2–4 µm and the overall dose of <20 e/Å^2.

Images were captured at multiple positions on each grid, and the diameters of the liposomes as well as the thickness of the lipid membranes were analysed using ImageJ software. It is worth noting that images also revealed unexpected structural complexity, including multilamellar vesicles (MLVs) and "nested" structures. However, such structural diversity is frequently observed in extruded liposomes, especially in vesicles with diameters exceeding 100 nm.[1]

1.6 Synchrotron small-angle X-ray scattering (SAXS) measurements

SAXS measurements were conducted at the SWING beamline at the SOLEIL synchrotron facility. In each experiment fresh samples were prepared with a lipid concentration of 6.4 mM to optimise scattering intensity. Before each experiment, a blank solution of 10.5 mM Na_2SO_4 was acquired and used as background. For each measurement, 7 mL of the liposome solution were placed in a vessel under stirring. The colloidal suspension was recirculated with a flow of 0.5 mL/min through a 1.5 mm thick quartz capillary using a peristaltic pump to conduct transmission SAXS data collection. A total of 100 frames were recorded for each sample with an exposure time of 2s per frame. Data processing, including background subtraction and averaging, was performed using the software Foxtrot-3.5.10.

For time-resolved SAXS (TR-SAXS) measurements, 100 frames were collected over 20 min. with a 2s exposure time per frame, and a 10s delay between each frame. The time between adding the corresponding reactant and following the safety protocols for closing the shutter and opening the X-ray beam varied between approximately 1.5 and 2.5 min.

The SAXS data were analysed using the software SASfit,[2] employing a model that combines a Gaussian bilayer form factor, represented by a sum of Gaussian functions to describe the electron density profile (Figure S29), with a modified Caillé structure factor tailored for lamellar systems. The SAXS parameters obtained from fitting the data are summarised in Table S1.

2. Chloride content inside liposomes

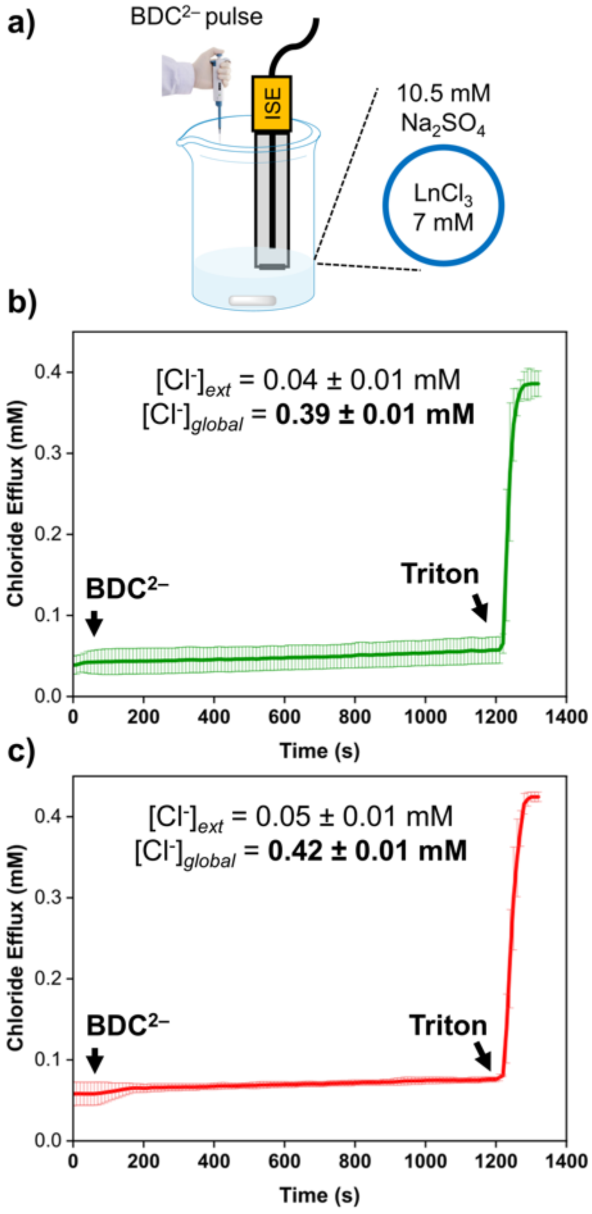

Figure S1. a Experimental setup for the Cl-ISE assay. Real-time chloride concentration measurements during the addition of BDC^{2-} and Triton X-100 to 3 mL solutions of **b** $TbCl_3$@LP and **c** $EuCl_3$@LP.

3. SEM-EDX analysis

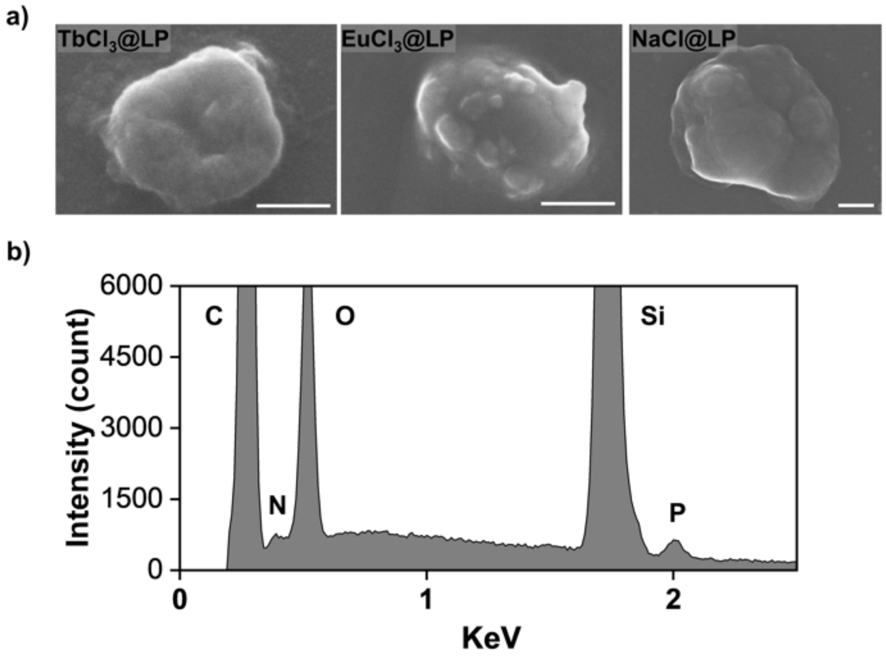

Figure S2. **a** SEM images of dried $TbCl_3$@LP, $EuCl_3$@LP, and NaCl@LP samples. Scale bar: 100 nm. **b** EDX analysis of the control sample NaCl@LP.

4. Fluorescence studies

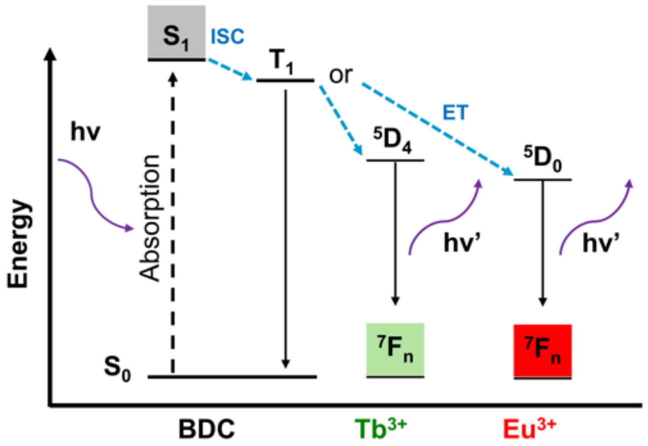

Figure S3. Schematic illustrating energy transfer from the T_1 excited states of the BDC^{2-} ligand to the $^5D_4$ excited state of Tb^{3+} and the $^5D_0$ excited state of Eu^{3+}, respectively.

Fluorescence studies of the TbBDC@LP system

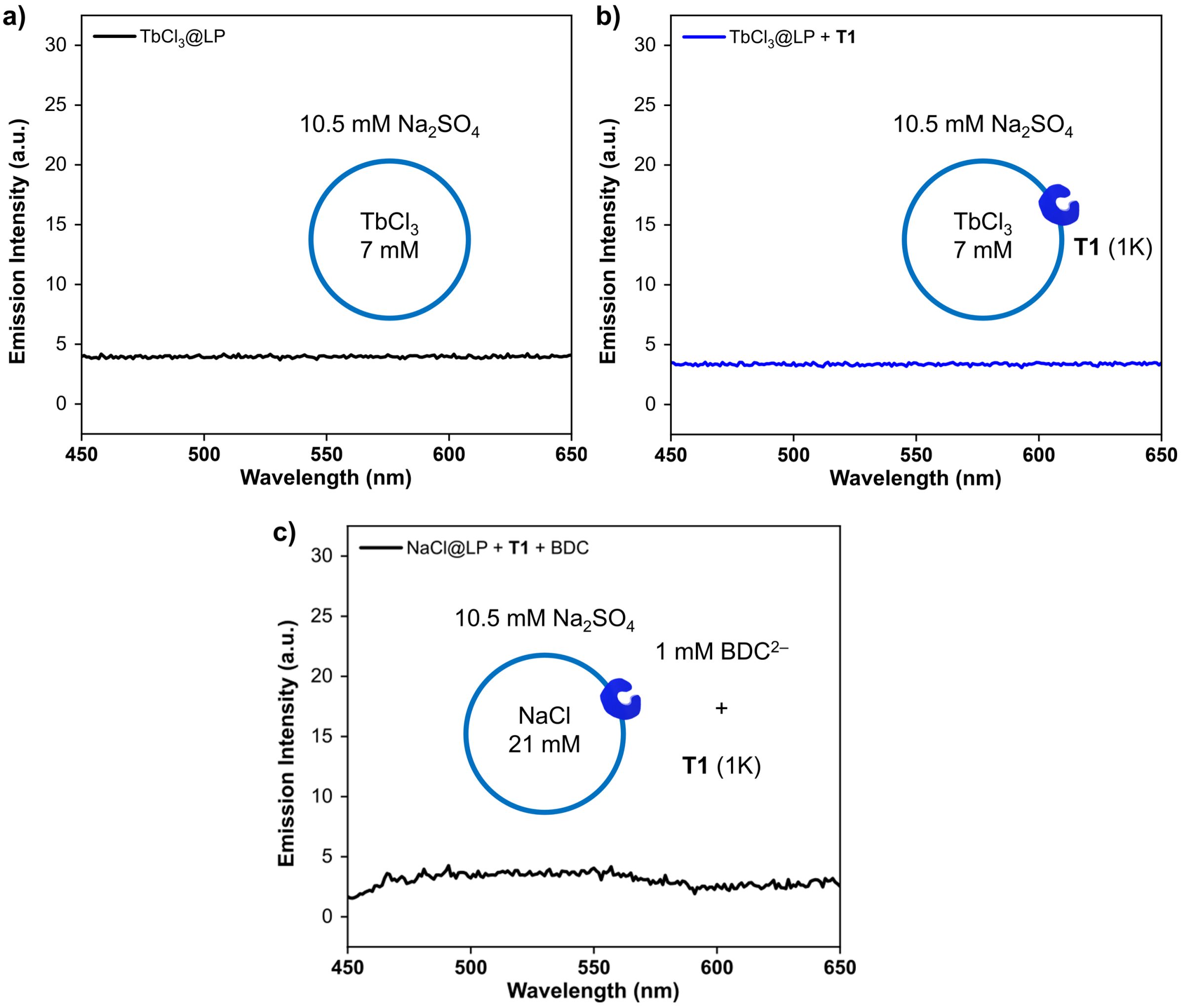

Figure S4. Emission spectra of 3 mL $TbCl_3$@LP samples in the **a** absence or **b** presence of transporter **T1** (1:1000), and **c** NaCl@LP in the presence of **T1** and BDC^{2-}, under 254 nm excitation.

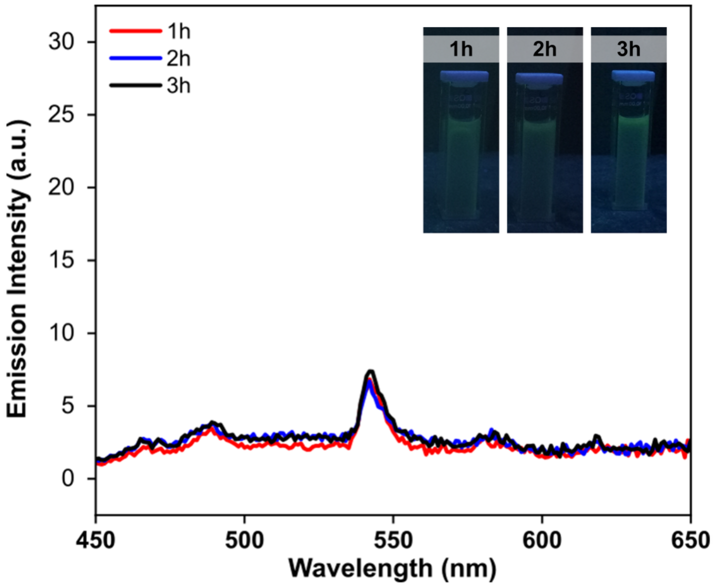

Figure S5. Emission spectra of the control sample ($TbCl_3$@LP + BDC^{2-}), monitoring for a period of 3 hours. Photographs showing emission under illumination with a handheld UV lamp (254 nm excitation) at 1, 2 and 3 hours after reactants addition.

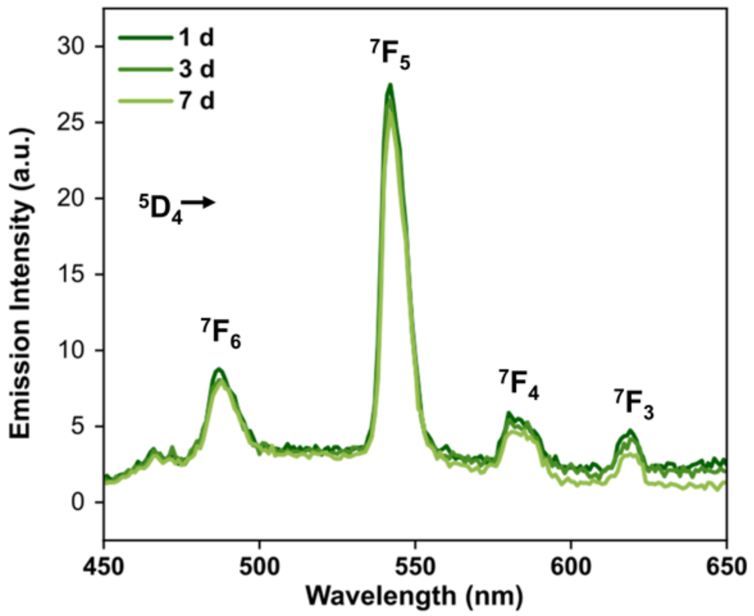

Figure S6. Emission spectra of the TbBDC@LP sample recorded after 1, 3 and 7 days of storage at room temperature.

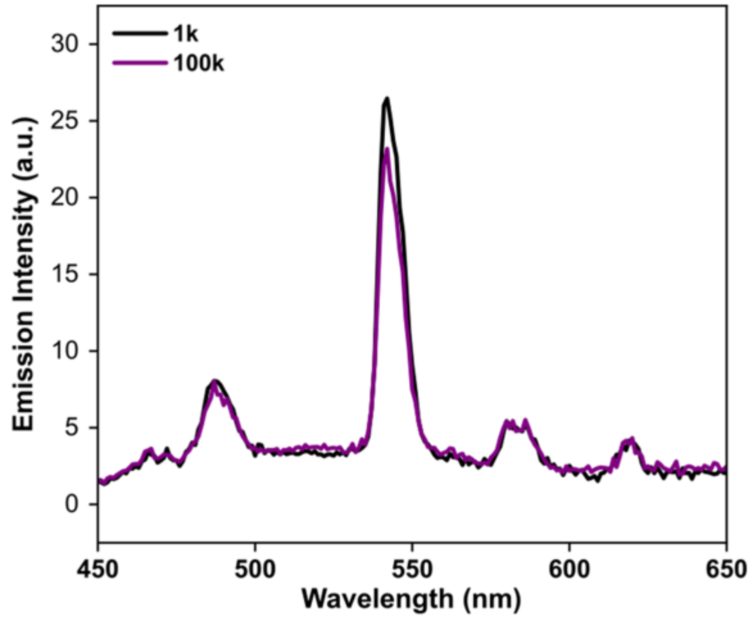

Figure S7. Emission spectra of TbBDC@LP samples with transporter concentrations at molar ratios of 1:1000 and 1:100,000, recorded 4h after preparation under 254 nm excitation.

Emission kinetics with delayed addition of BDC^{2-}

To test whether the presence of **T1** itself might promote Tb^{3+} leakage, we performed a pulse experiment in which BDC^{2-} was added 1 hour after **T1** incorporation. The resulting emission kinetics were comparable to those in the standard setup, suggesting that metal ion retention is not compromised by the presence of **T1** (Figure S8).

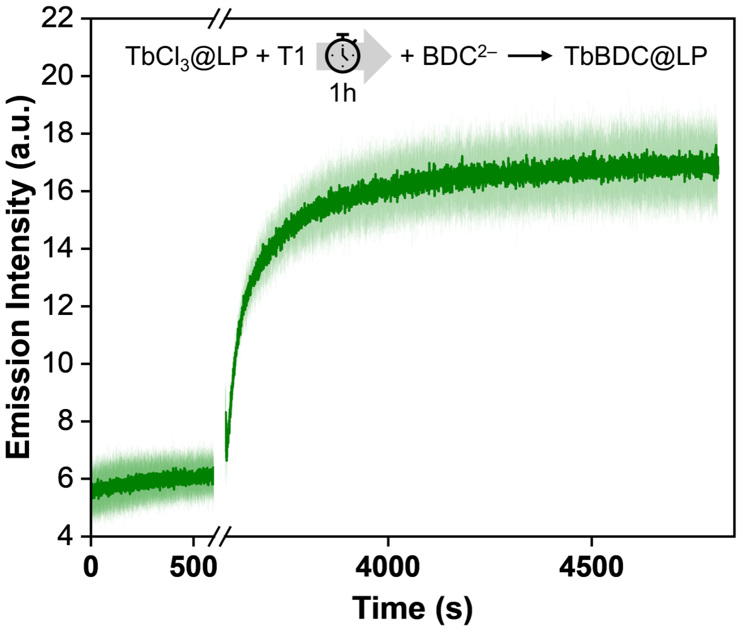

Figure S8. Emission kinetics with a delayed addition of BDC^{2-}.

Dialysis assay to test for leakage of Tb^{3+} or Tb(BDC) complexes post-formation

To discard a potential leakage of Tb^{3+} or Tb(BDC) complexes post-formation, a TbBDC@LP solution was subjected to a 24-hour dialysis experiment against an equal volume of 10.5 mM Na_2SO_4. A dialysis membrane separated the two solutions (Figure S9a). No characteristic emission bands were detected in the external sulphate phase. Moreover, no emission was observed even after the addition of BDC^{2-} to the sulphate phase, confirming the absence of leached Tb^{3+} or formed Tb(BDC) complexes (Figure S9b).

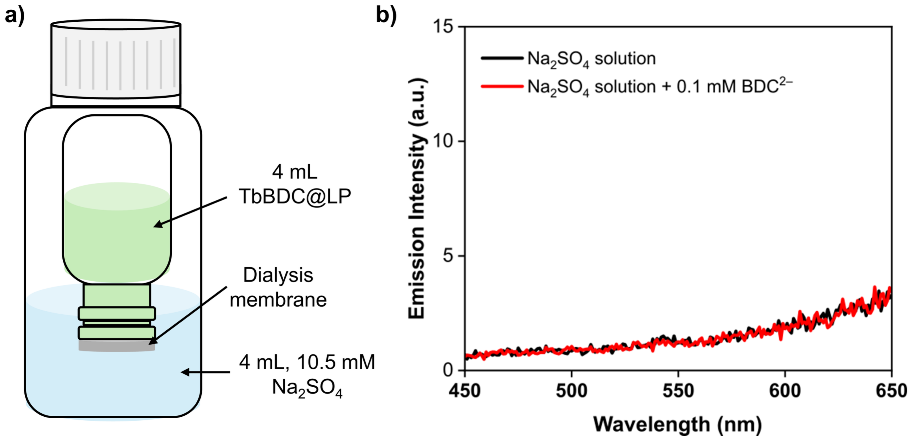

Figure S9: **a** Dialysis assay to test the potential leakage of Tb^{3+} or Tb(BDC) complexes post-formation of the TbBDC@LP system. **b** Emission spectra of the external Na_2SO_4 solution.

Coordination reaction between Tb^{3+} and BDC^{2-} in aqueous solution

To evaluate the coordination reaction between Tb^{3+} and BDC^{2-} in the absence of liposomes, we conducted a reaction using 7 mM $TbCl_3$ and 10.5 mM BDC^{2-}, concentrations comparable to those estimated inside liposomes. As expected for the formation of the insoluble coordination polymer Tb_2BDC_3, the product rapidly precipitated in aqueous solution (Figure S10a). Additionally, we recorded the emission spectrum of a reaction using lower concentrations (0.13 mM $TbCl_3$ with 0.2 mM BDC^{2-}), consistent with the levels measured using the Cl-ISE electrode. In this case, characteristic terbium emission bands were observed. However, the product gradually precipitated over time within the quartz cuvette.
Kinetic experiments under these concentrations were not feasible, as the emission intensity rapidly exceeded the limit of the detector, risking damage to the instruments.

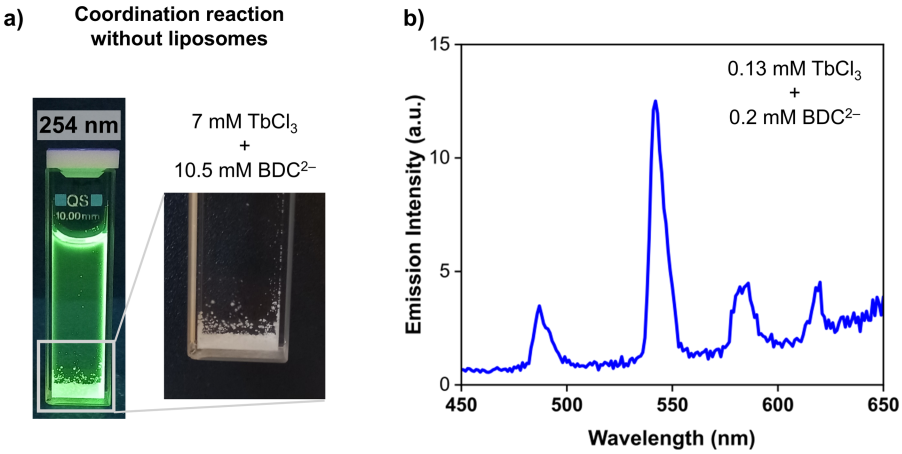

Figure S10: **a** Results of the coordination reaction between Tb^{3+} and BDC^{2-} in aqueous solution, showing the rapid precipitation of the product. Photography of the sample under illumination with a handheld UV lamp (254 nm excitation). **b** emission spectrum from a lower concentration reaction: 0.13 mM $TbCl_3$ with 0.2 mM BDC^{2-}.

Formation of the TbBDC@LP system using different lipid compositions

To evaluate the generality of our system across different lipid compositions, we prepared TbBDC@LP systems using liposomes composed entirely of POPC (Figure S11) and DOPC (Figure S12), in addition to the POPC/cholesterol mixture (7:3) used in the main text. DLS profiles, emission spectra, and formation kinetics at a transporter-to-lipid ratio of 1:1000 confirm that luminescent complex formation is not limited to a specific lipid composition.

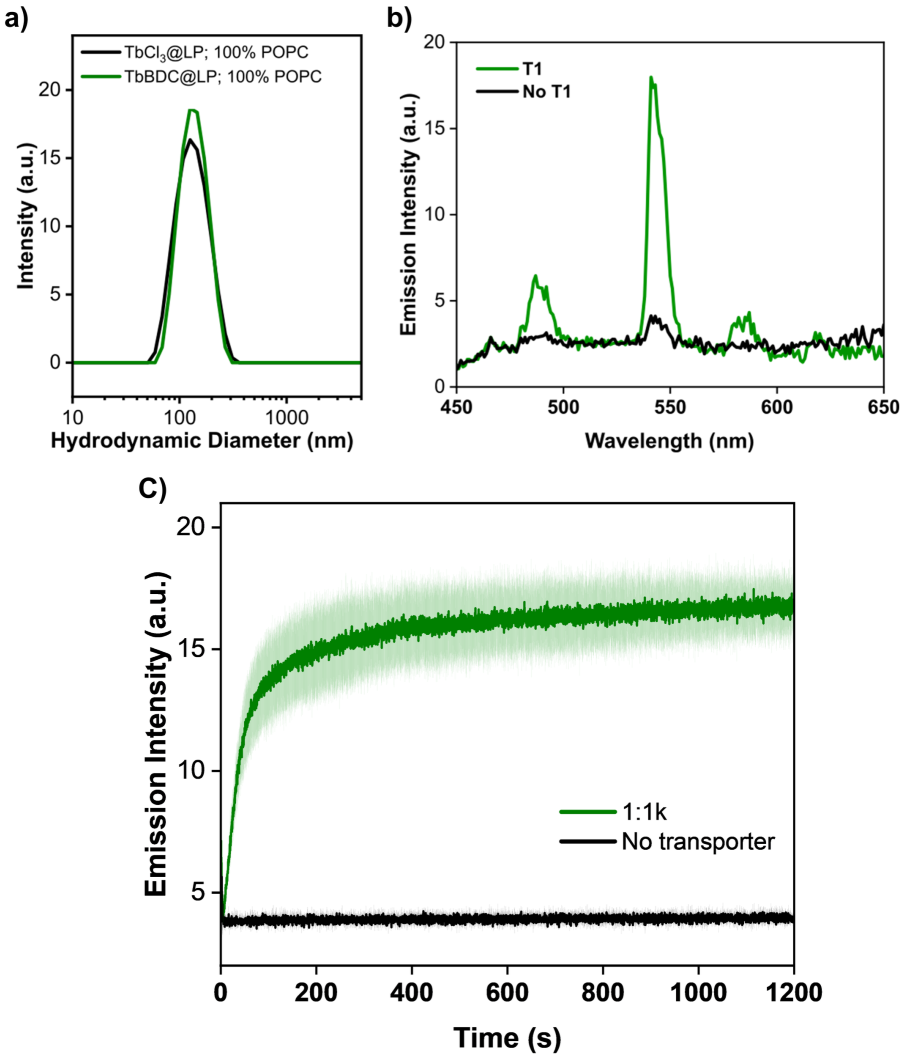

Figure S11: Formation of the TbBDC@LP system using liposomes composed entirely of POPC. **a** DLS measurements confirming a monodisperse size distribution of the vesicles. **b** Emission spectra comparing the TbBDC@LP system and the control sample ($TbCl_3$@LP + BDC^{2-}) upon excited at λ_{ex} = 254 nm. **c** The emission intensity of LnBDC@LP system over time at a transporter-to-lipid molar ratio of 1:1000.

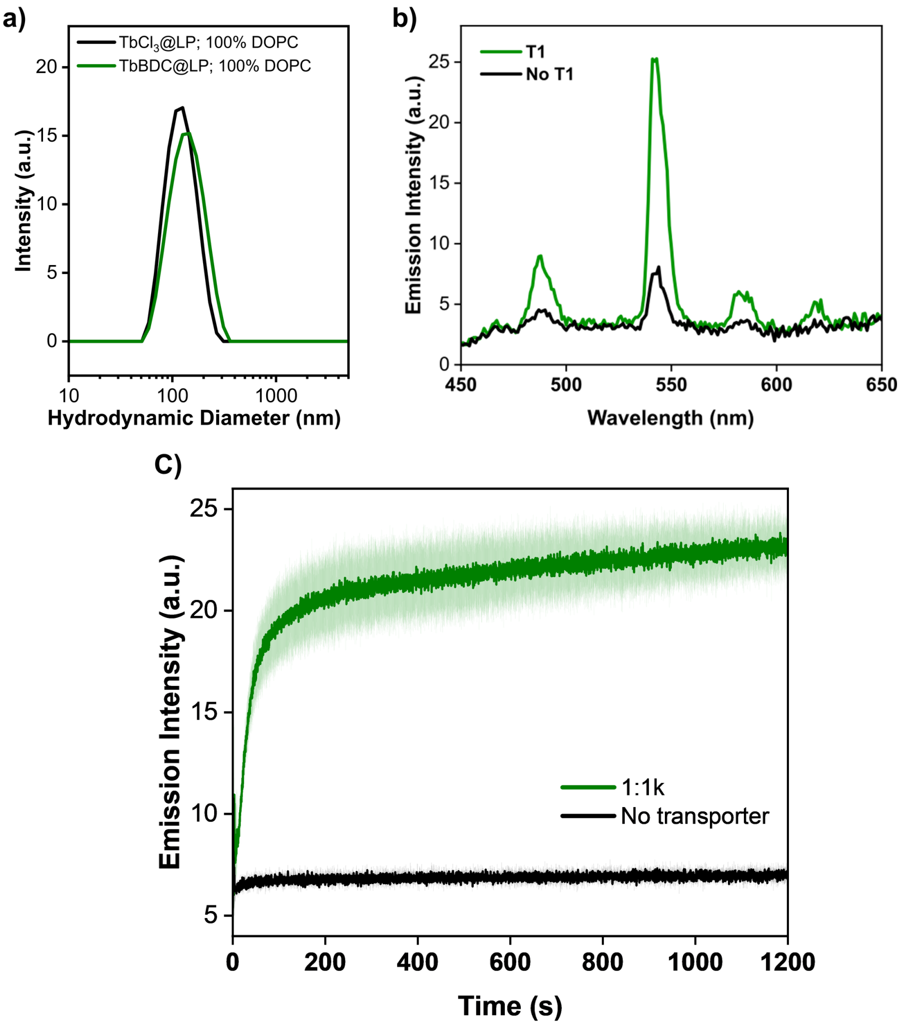

Figure S12: Formation of the TbBDC@LP system using liposomes composed entirely of DOPC. **a** DLS measurements confirming a monodisperse size distribution of the vesicles. **b** Emission spectra comparing the TbBDC@LP system and the control sample ($TbCl_3$@LP + BDC^{2-}) upon excited at λ_{ex} = 254 nm. **c** The emission intensity of LnBDC@LP system over time at a transporter-to-lipid molar ratio of 1:1000.

Fluorescence studies of the EuBDC@LP system

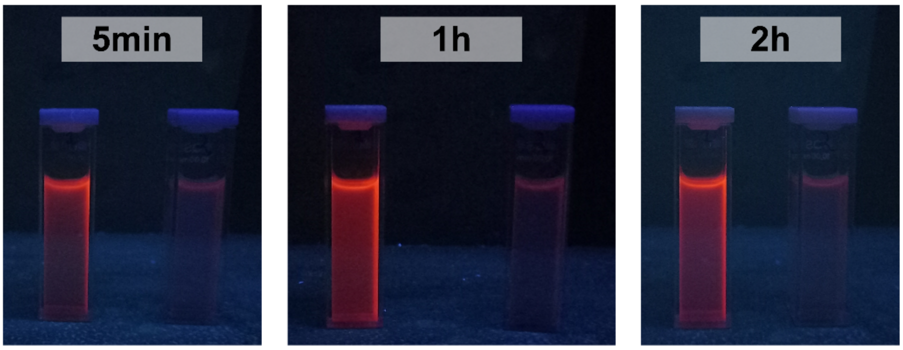

Figure S13. Photographs showing emission differences between samples EuBDC@LP ($EuCl_3$@LP + BDC^{2-} + **T1**; left) and $EuCl_3$@LP + BDC^{2-} (right), under handheld UV lamp illumination (254 nm excitation) at 5 min., 1h, and 2h after reactants addition.

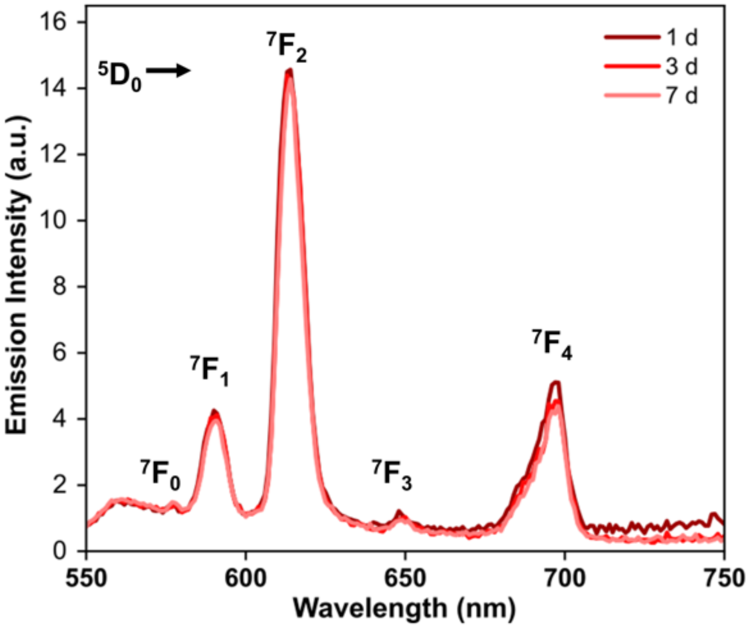

Figure S14. Emission spectra of the EuBDC@LP sample recorded after 1, 3 and 7 days of storage at room temperature.

5. FT-IR spectroscopy

To further confirm the coordination between terbium and BDC^{2-}, FT-IR spectroscopy was performed, replacing the sulphate solution with a 21 mM NaCl solution to avoid interference from SO_4^{2-} absorption peaks. A dried TbBDC@LP sample displayed an asymmetric $RCOO^-$ peak at 1567 cm^{-1}, distinct from the Na_2BDC peak at 1553 cm^{-1} and the 1676 cm^{-1} peak of protonated H_2BDC acid, confirming carboxylate coordination to Tb^{3+}.

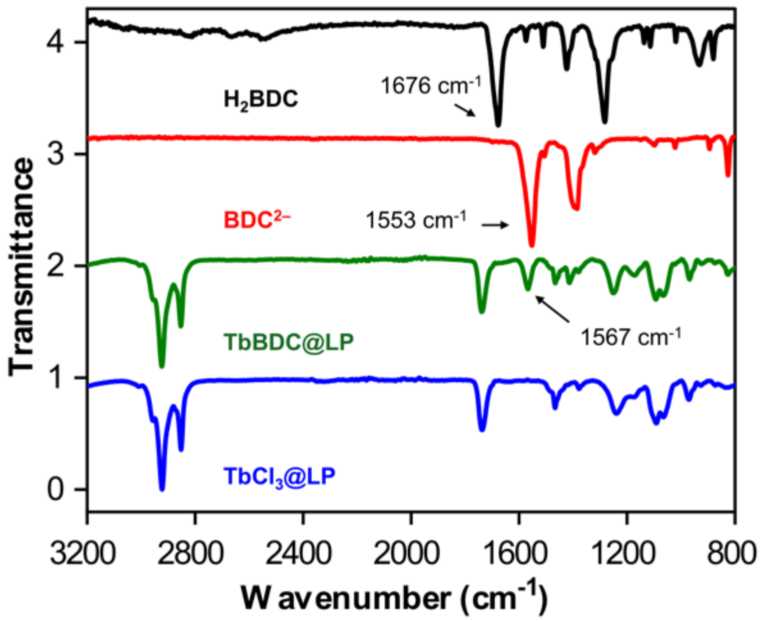

Figure S15. FT-IR spectra comparison of TbBDC@LP, $TbCl_3$@LP, 1,4-benzenedicarboxylate sodium salt (BDC^{2-}), and 1,4-benzenedicarboxylic acid. The spectra highlight the asymmetric $RCOO^-$ peaks.

6. Cryo-TEM analysis

For the size histogram analysis, unilamellar, multilamellar, and multivesicular vesicles were included in the analysis, provided they were spherical or near-spherical vesicles with fully distinguishable and complete diameters. For the $TbCl_3$@LP samples, measurements were taken form 67 vesicles, while for the other samples, more than 100 vesicles were analysed.

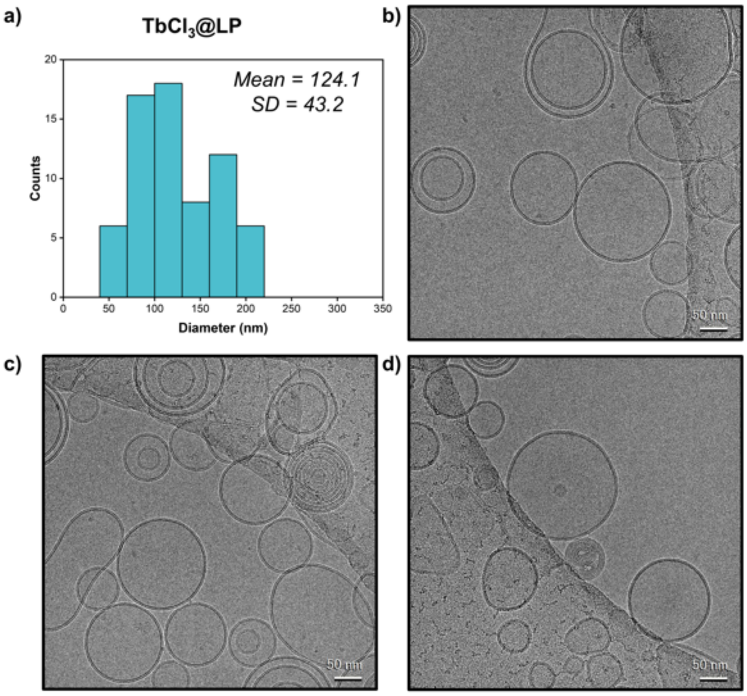

Figure S16. a Diameter distribution histrogram of $TbCl_3$@LP. **b–d** TEM images of liposomes captured at different position on the grid, showing predominantly unilamellar vesicles.

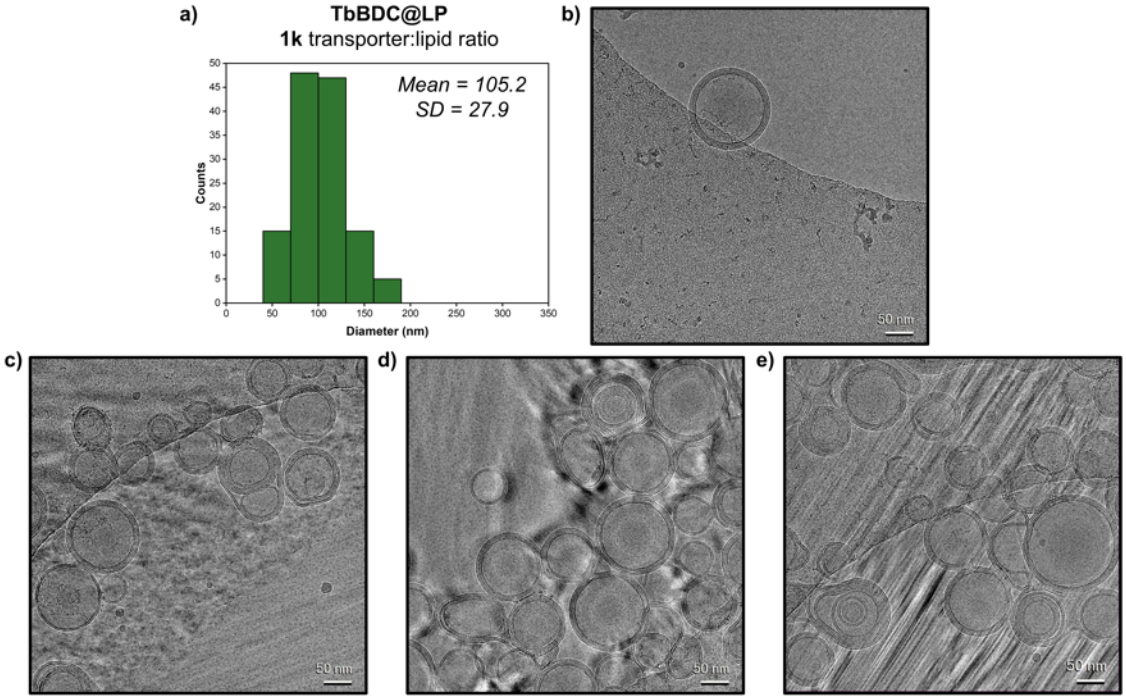

Figure S17. a Diameter distribution histrogram of TbBDC@LP with a transporter:lipid ratio of 1:1,000. **b–e** TEM images of liposomes captured at different position on the grid, showing predominantly bilamellar vesicles.

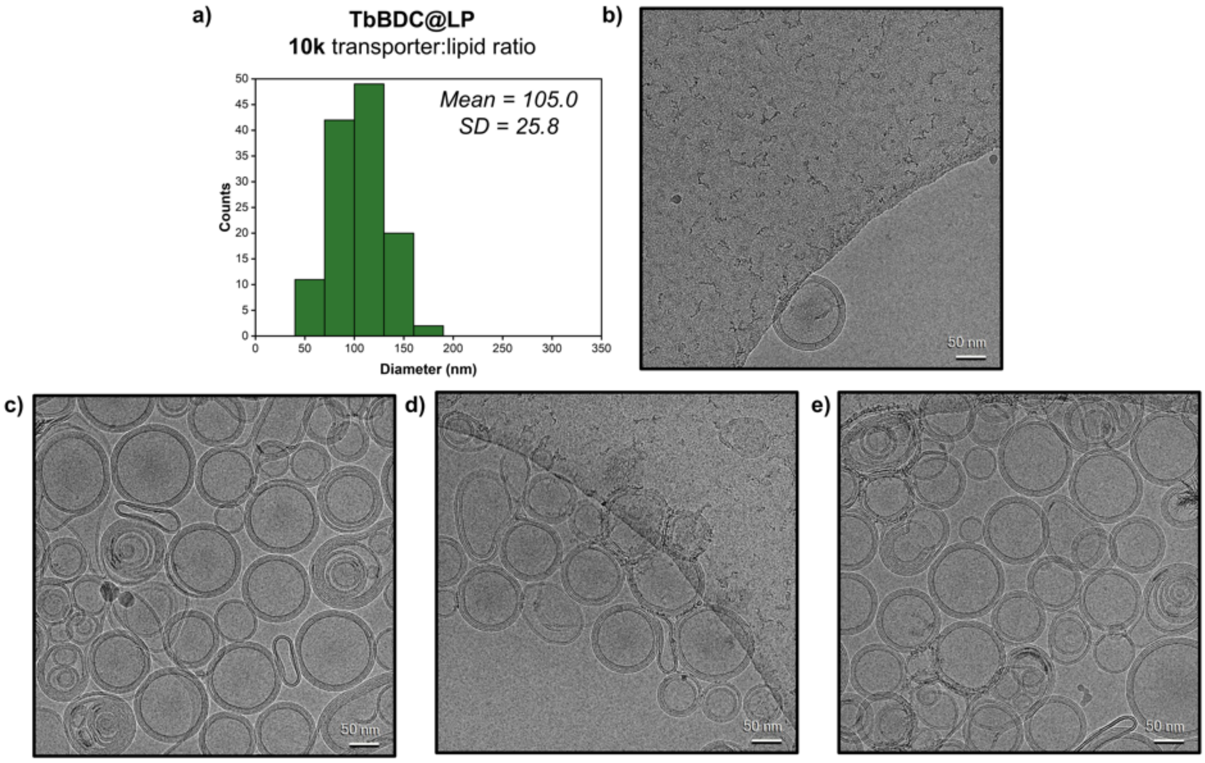

Figure S18. a Diameter distribution histrogram of TbBDC@LP with a transporter:lipid ratio of 1:10,000. **b**–**e** TEM images of liposomes captured at different position on the grid, showing predominantly bilamellar vesicles.

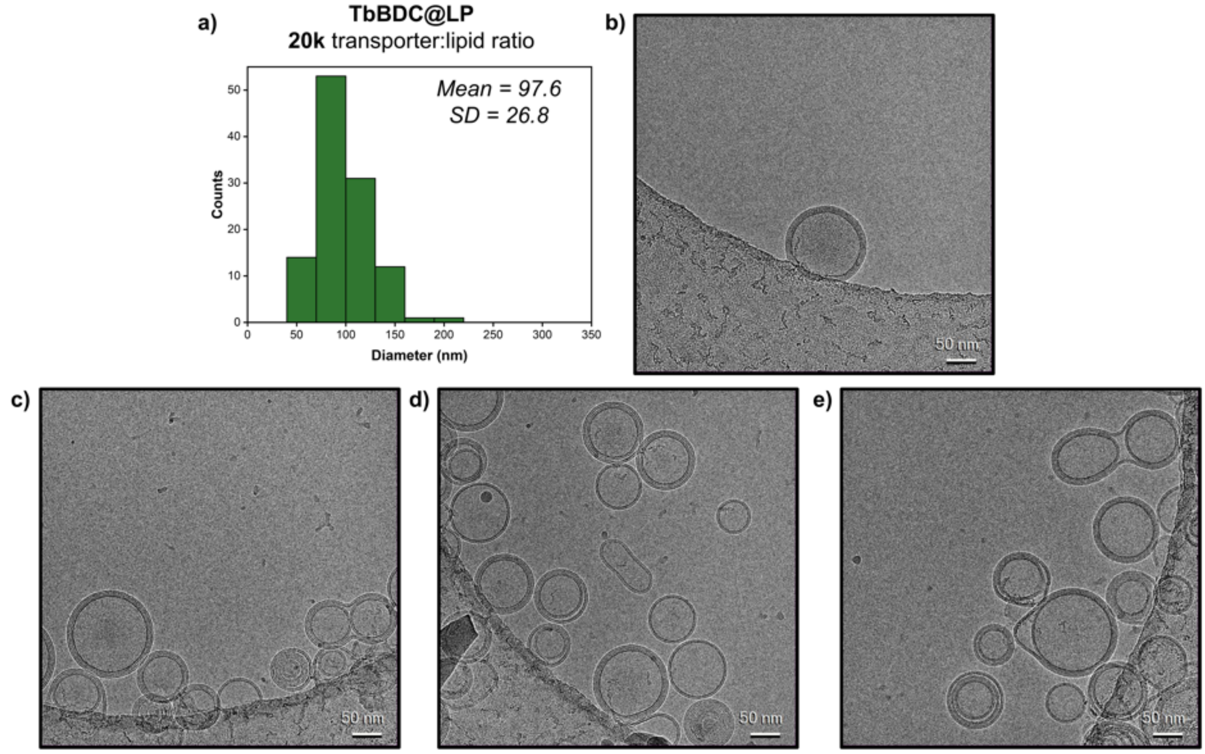

Figure S19. a Diameter distribution histrogram of TbBDC@LP with a transporter:lipid ratio of 1:20,000. **b**–**e** TEM images of liposomes captured at different position on the grid, showing predominantly bilamellar vesicles.

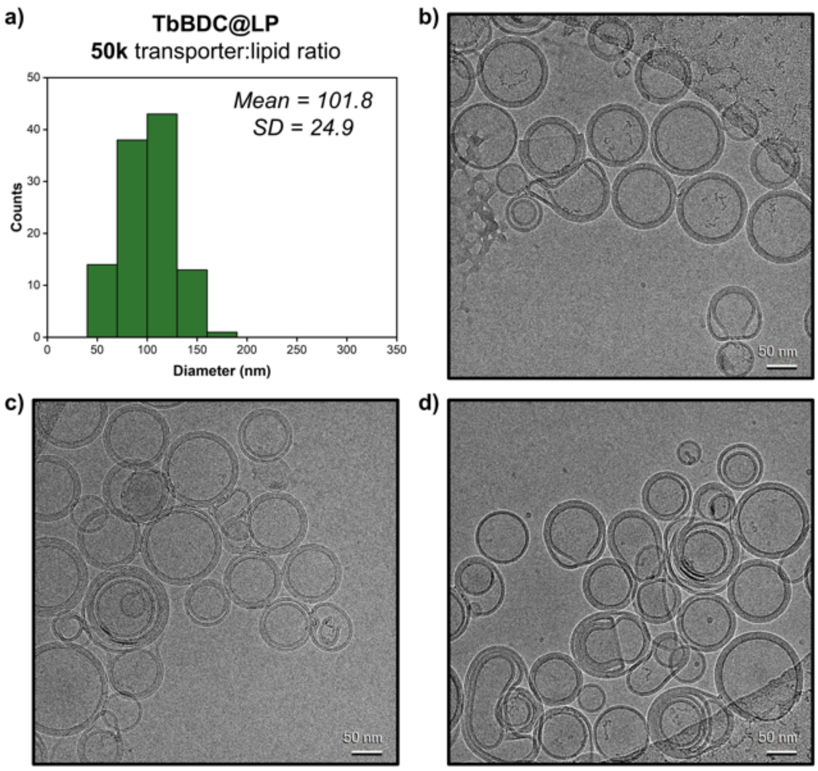

Figure S20. a Diameter distribution histrogram of TbBDC@LP with a transporter:lipid ratio of 1:50,000. **b**–**d** TEM images of liposomes captured at different position on the grid, showing predominantly bilamellar vesicles.

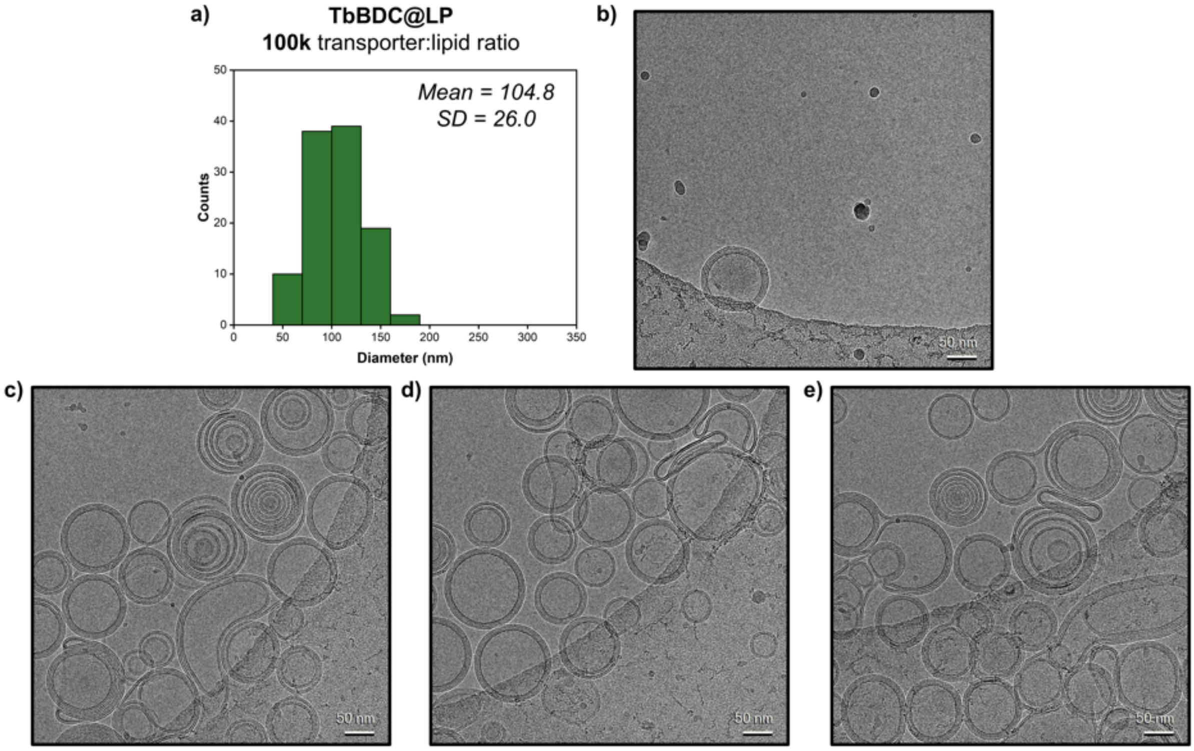

Figure S21. a Diameter distribution histrogram of TbBDC@LP with a transporter:lipid ratio of 1:100,000. **b**–**e** TEM images of liposomes captured at different position on the grid, showing predominantly bilamellar vesicles.

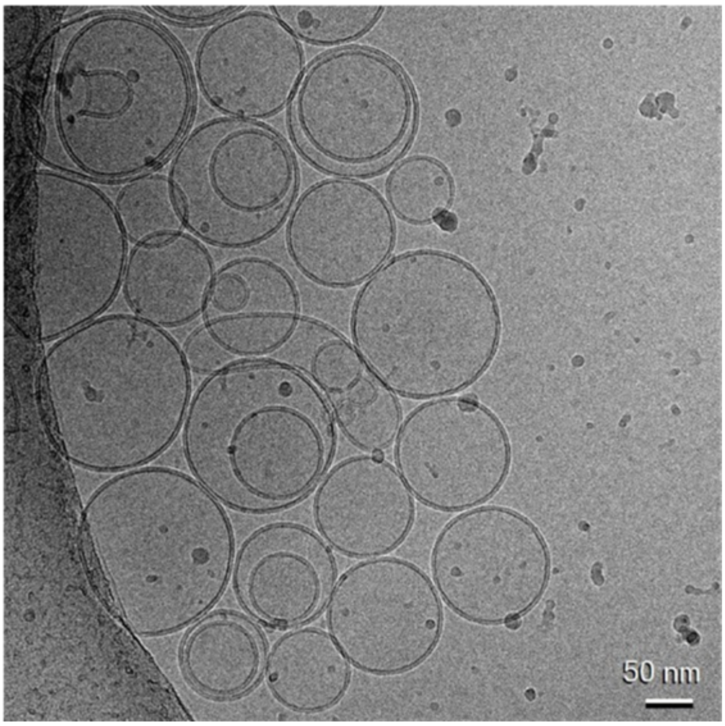

Figure S22.TEM images of control sample NaCl@LP + **T1** + BDC^{2-}.

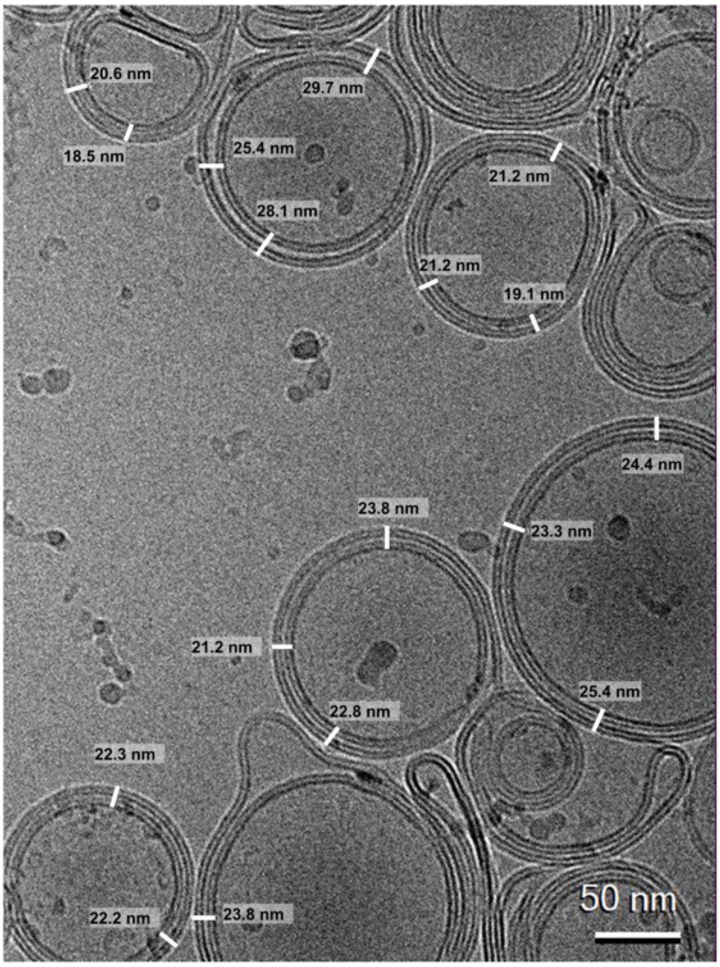

Figure S23. TEM image of EuBDC@LP.

Formation of bilamellar vesicles via terbium-succinate complexation in liposome-based nanoreactors

We also explored the use of an alternative dicarboxylate, specifically, the aliphatic succinate, to form the TbSuccinate@LP system. The same protocol used for BDC was applied, employing a lipid composition of POPC:Cholesterol in a 7:3 molar ratio. DLS measurements confirmed a monodisperse size distribution and colloidal stability in water (Figure S24a). As expected for aliphatic carboxylates, only negligible emission was observed (Figure S24b). Interestingly, cryo-TEM analysis revealed the formation of bilamellar vesicles (Figure S24c), consistent with our previous results using BDC. This finding suggest that the structural transformation to bilamellar vesicles is not limited to aromatic ligands and highlights the potential for tuning vesicles architecture via coordination chemistry, broadening the scope of applications in controlled double-membrane formation.

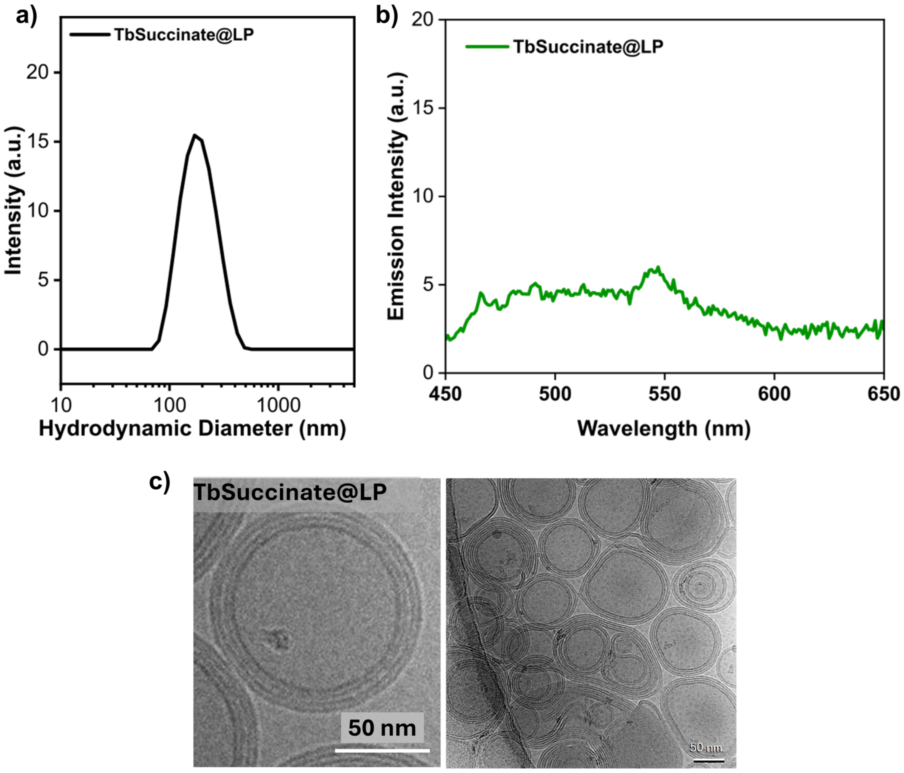

Figure S24: a) DLS of the TbSuccinate@LP system. a) Emission spectrum of the TbSuccinate@LP system, showing negligible emission. c) Cryo-TEM image revealing the formation of bilamellar vesicles.

7. Estimation of the size of bilamellar vesicles

To calculate the diameter of the resulting bilamellar vesicles, we assumed that the original vesicle has a surface area S_i, while the spherical bilamellar vesicles have a surface area S_f that is approximately half of S_i:

$$S_f = \frac{S_i}{2} \qquad \text{Eq. S1}$$

Expressing the surface areas as functions of their respective sphere radii, we have:

$$S_i = 4\pi r_i^2 \text{ and } S_f = 4\pi r_f^2 \qquad \text{Eq. S2}$$

Simplifying these equations, the relationship between the radii of the bilamellar vesicles (r_f) and the original vesicle (r_i) becomes:

$$r_f = \frac{r_i}{\sqrt{2}} \qquad \text{Eq. S3}$$

Using the average radius obtained from cryo-TEM for $TbCl_3$@LP, which is 62 nm, we calculated a theoretical radius of 44 nm. This value is close to the experimental mean of 51 nm measured across all TbBDC@LP samples. However, it should be noted that the experimental radius is slightly higher due to additional factors not considered in this approximation. These factors include the reduced number of lipids required to form the internal sphere of the bilamellar vesicle and the interlayer space between the two bilayers, both of which contribute to an increase in the observed radius.

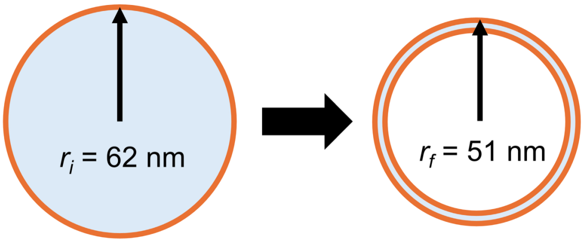

Figure S25. Radius measurements for $TbCl_3$@LP (left) and TbBDC@LP (right) samples.

8. Synchrotron small-angle X-ray scattering (SAXS) measurements

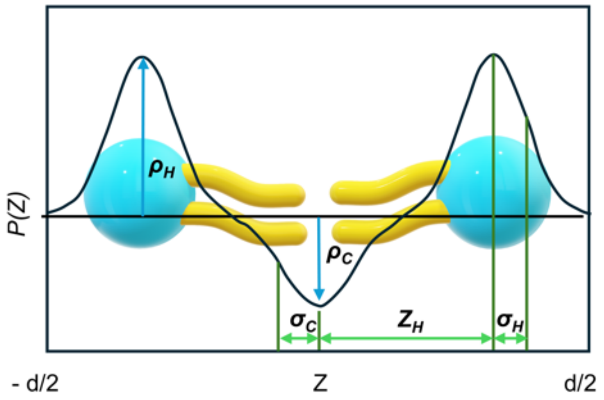

Figure S26. Gaussian model of the electron density profile $\rho(Z)$ as a function of distance Z from the centre of the bilayer.

Where:
Z_H = Distance of the headgroup to the bilayer centre
σ_H = Gaussian half-width for outer layer surface
ρ_H = Scattering contrast for headgroup
σ_C = Gaussian half-width for inner layer
ρ_C = Scattering contrast for inner layer

Table S1 | SAXS parameters obtained from fitting the experimental data.

Sample		Form factor					Structure factor		
		d_{HH} ($2Z_H$) [Å]	σ_H [Å]	ρ_H [cm^{-1}]	σ_C [Å]	ρ_C [cm^{-1}]	N[a] [a. u.]	d_L[b] [Å]	η[c] [a. u.]
**$TbCl_3$@LP**		41	3.6	0.052	4.9	-0.061	1.1	75	0.2
**TbBDC@LP**	Part I (85.7%)	40	3.6	0.052	4.9	-0.05	2.3	68	0.12
	Part II (14.3%)	40	3.6	0.015	4.9	-0.13	2.3	68	0.12
**TbBDC@LP** (105 s)		40	3.6	0.035	4.9	-0.064	1.2	70	0.8
**TbBDC@LP** (129 s)		40	3.6	0.035	4.9	-0.064	1.25	70	0.6
**TbBDC@LP** (153 s)		39.5	3.6	0.034	4.9	-0.064	1.35	72	0.4
**TbBDC@LP** (189 s)		39	3.6	0.031	4.9	-0.059	1.55	70	0.22
**TbBDC@LP** (237 s)		41	3.6	0.026	4.9	-0.051	1.85	69	0.26
**TbBDC@LP** (369 s)		41	3.6	0.04	4.9	-0.08	2.1	68	0.18
**TbBDC@LP** (1293 s)		40.5	3.6	0.037	4.9	-0.078	2.3	68	0.16
**$EuCl_3$@LP**		41	3.6	0.037	4.8	-0.078	1.13	68.5	0.17
**EuBDC@LP**	Part I (85%)	40.5	3.6	0.05	4.9	-0.052	2.2	69.5	0.2
	Part II (15%)	41	3.6	0.01	4.9	-0.136	2.2	69	0.2

[a] Number of layers (lamellarity). [b] Layer spacing. [c] Caillé parameter

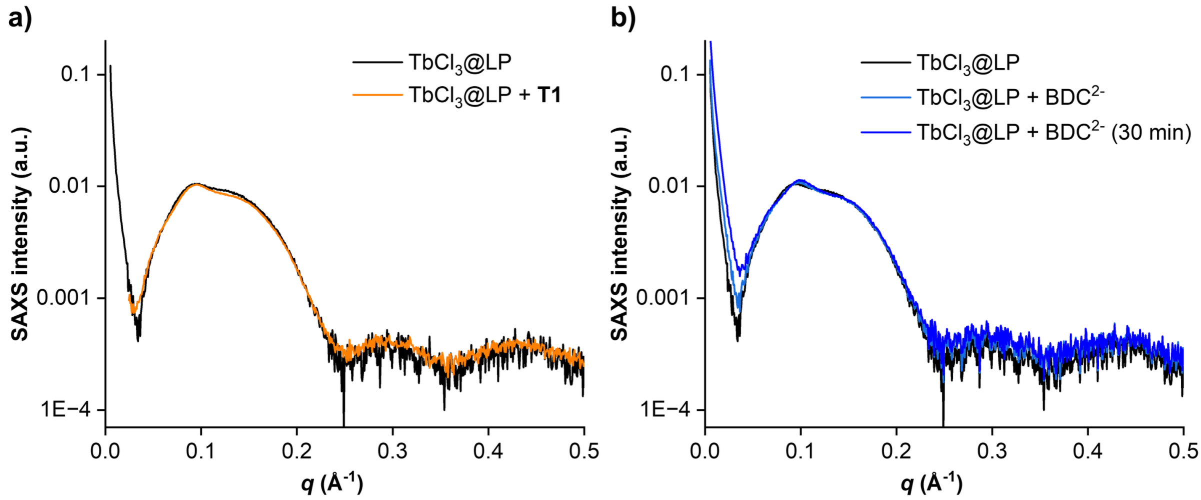

Figure S27: **a** SAXS profile of $TbCl_3$@LP before (black) and after (orange) the addition of **T1**. **b** SAXS profile of $TbCl_3$@LP before (black) and after (light blue) the addition of BDC^{2-}. An additional acquisition taken 30 min. after BDC^{2-} addition (dark blue) shows no significant changes in the scattering profile.

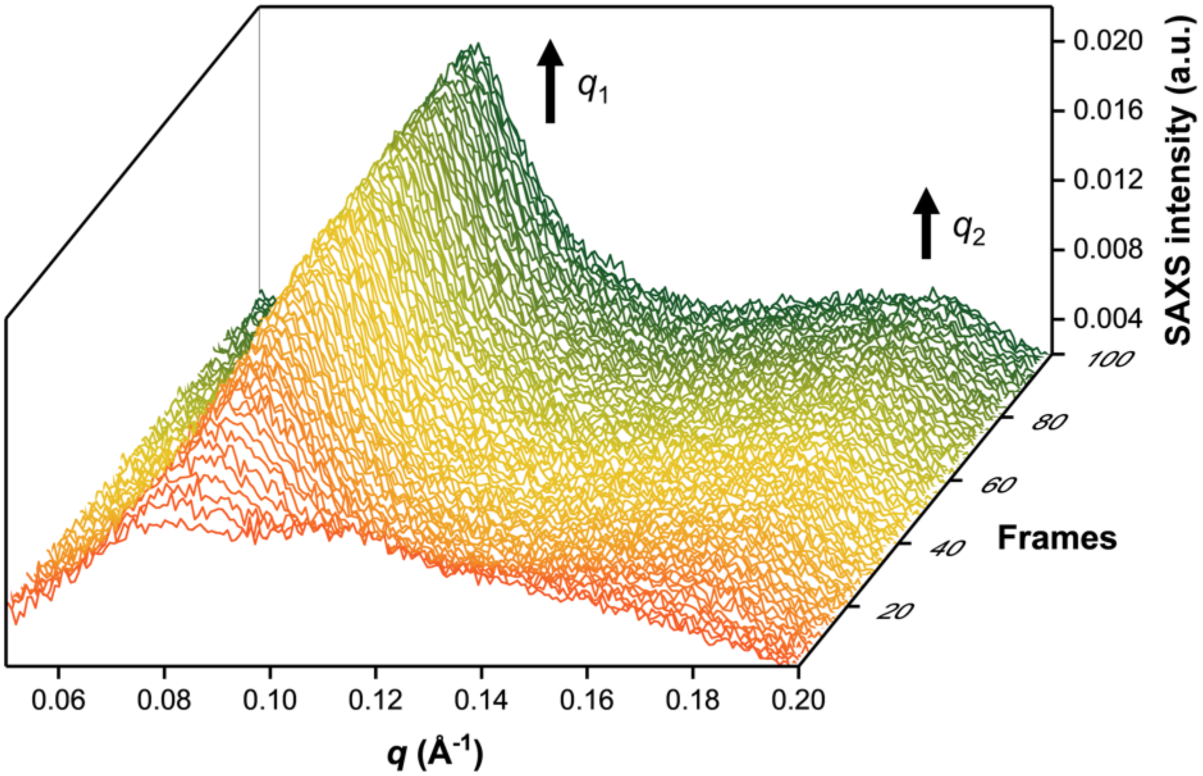

Figure S28. 3D representation of time-resolved SAXS data showing 100 frames acquired over 20 min. to monitor the formation of TbBDC@LP though the sequence: (1) $TbCl_3$@Liposome + (2) **T1** (1k) + (3) BDC^{2-}. The intensity of two broad peaks at q_1 = 0.091 and q_2 = 0.180 Å^{-1} increase over time, indicating the formation of bilamellar vesicles. The first frame was collected 93s after adding BDC^{2-}.

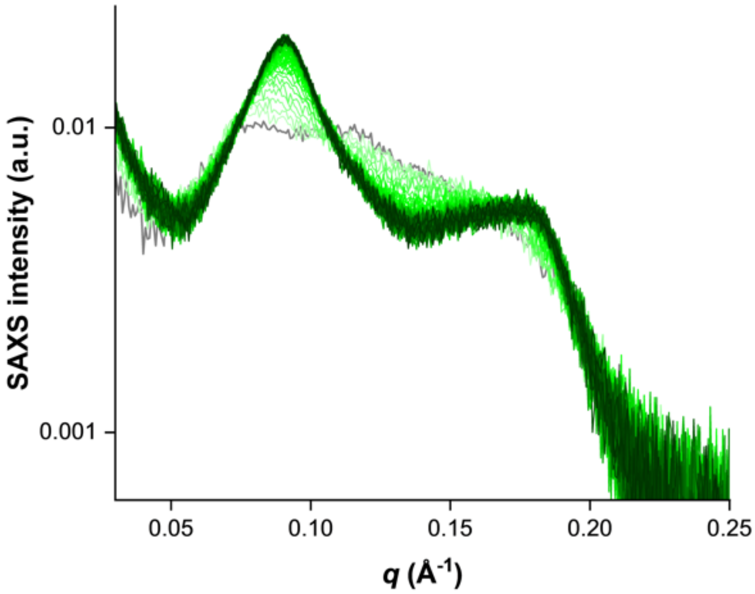

Figure S29. 2D representation of time-resolved SAXS data showing 100 frames acquired over 20 min. to monitor the formation of TbBDC@LP though the sequence: (1) $TbCl_3$@LP + (2) **T1** (1k) + (3) BDC^{2-}.

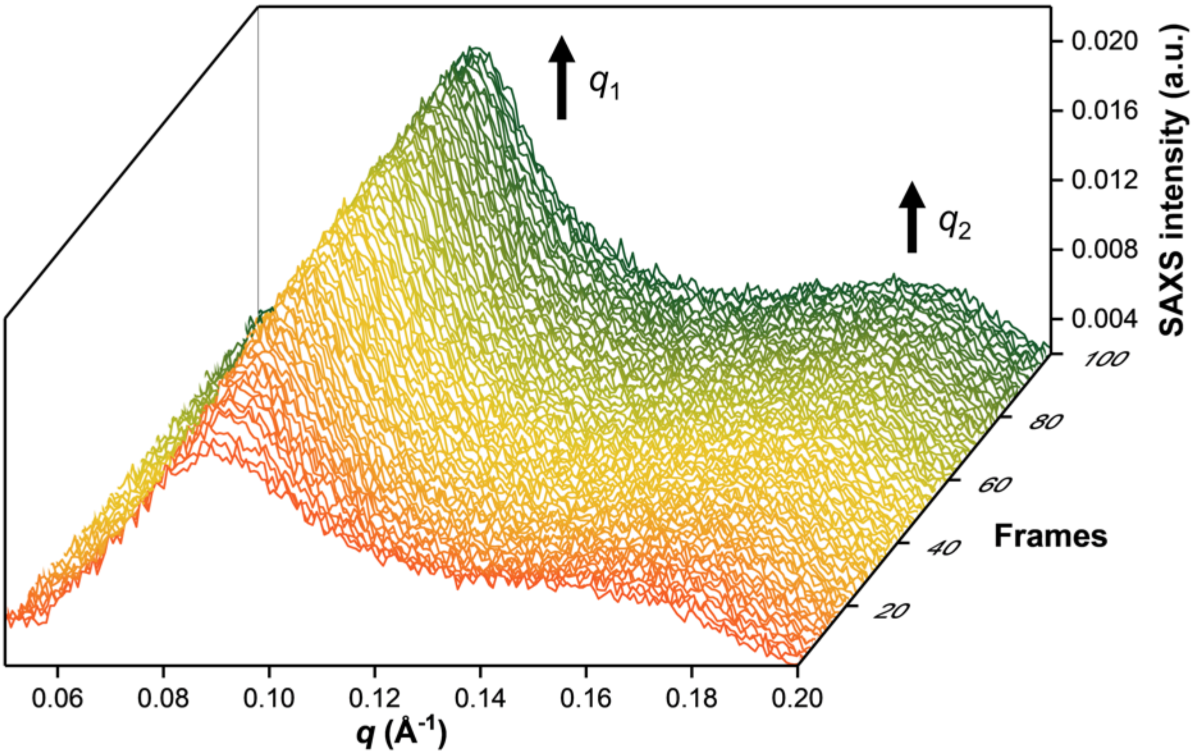

Figure S30. 3D representation of time-resolved SAXS data showing 100 frames acquired over 20 min. to monitor the formation of TbBDC@LP though the sequence: (1) $TbCl_3$@LP + (2) BDC^{2-} + (3) **T1** (1k). The first frame was collected 152s after adding BDC^{2-}.

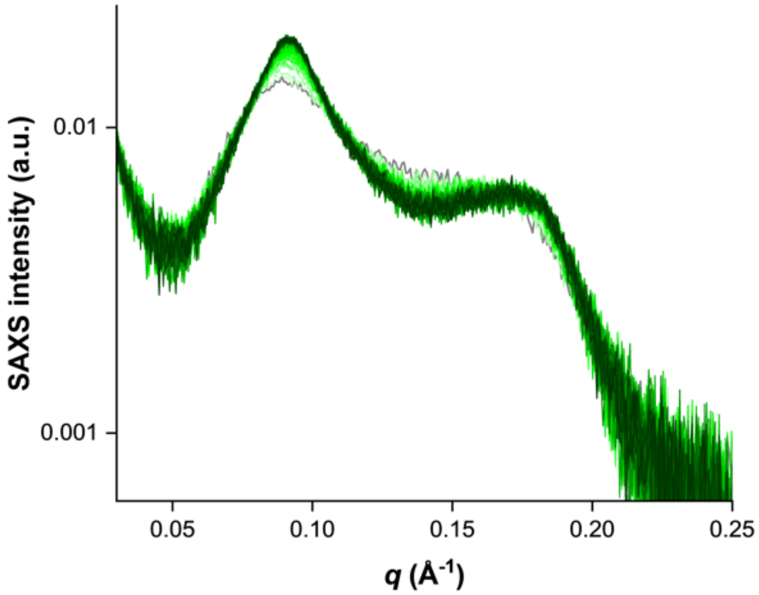

Figure S31. 2D representation of time-resolved SAXS data showing 100 frames acquired over 20 min. to monitor the formation of TbBDC@LP though the sequence: (1) $TbCl_3$@LP + (2) BDC^{2-} + (3) **T1** (1k).

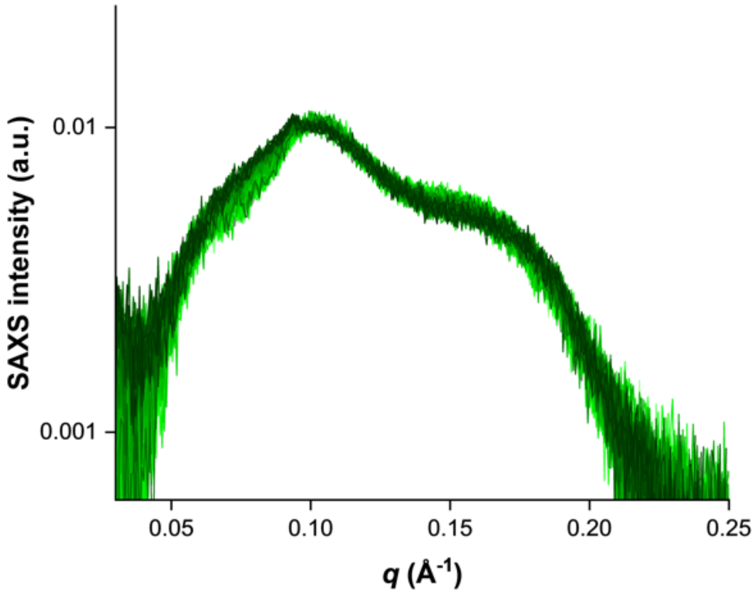

Figure S32. 2D representation of time-resolved SAXS data showing 100 frames acquired over 20 min. to monitor the formation of TbBDC@LP though the sequence: (1) $TbCl_3$@LP + (2) BDC^{2-} + (3) **T1**, using a transport:lipid ratio of 1:50,000. The first frame was collected 118s after adding BDC^{2-}.

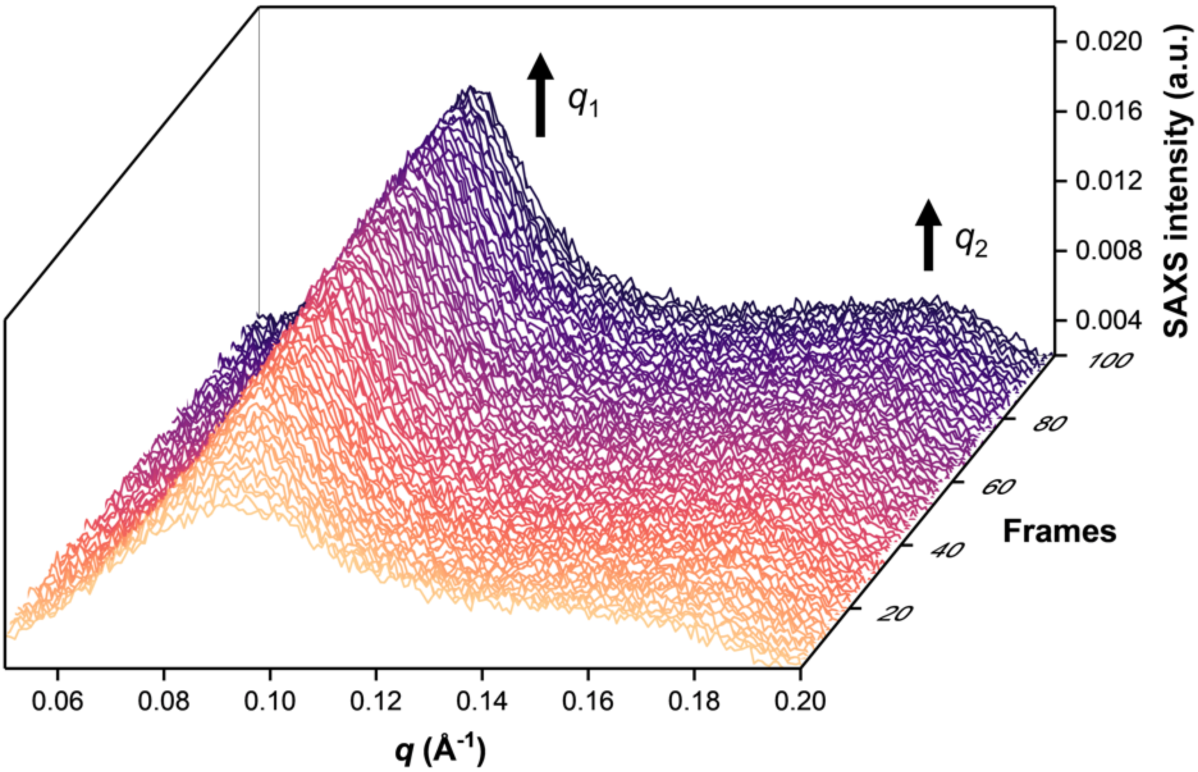

Figure S33. 3D representation of time-resolved SAXS data showing 100 frames acquired over 20 min. to monitor the formation of EuBDC@LP though the sequence: (1) $EuCl_3$@LP + (2) BDC^{2-} (3) **T1** (1k). The intensity of two broad peaks at q_1 = 0.091 and q_2 = 0.180 $Å^{-1}$ increase over time, indicating the formation of bilamellar vesicles. The first frame was collected 146s after adding BDC^{2-}.

References

[1] H. L. Scott, A. Skinkle, E. G. Kelley, M. N. Waxham, I. Levental, F. A. Heberle, *Biophysical Journal* **2019**, *117*, 1381–1386.

[2] I. Breßler, J. Kohlbrecher, A. F. Thünemann, *J. Appl. Crystallogr.* **2015**, *48*, 1587–1598.
